# Long noncoding RNA DIO3OS induces glycolytic-dominant metabolic reprogramming to promote aromatase inhibitor resistance in breast cancer

**DOI:** 10.1038/s41467-022-34702-x

**Published:** 2022-11-22

**Authors:** Xueman Chen, Rong Luo, Yunmei Zhang, Shuying Ye, Xin Zeng, Jiang Liu, Di Huang, Yujie Liu, Qiang Liu, Man-Li Luo, Erwei Song

**Affiliations:** 1 Guangdong Provincial Key Laboratory of Malignant Tumor Epigenetics and Gene Regulation, Guangdong-Hong Kong Joint Laboratory for RNA Medicine, Sun Yat-Sen Memorial Hospital, Sun Yat-Sen University, Guangzhou, China; 2 Breast Tumor Center, Sun Yat-Sen Memorial Hospital, Sun Yat-Sen University, Guangzhou, China; 3 Program of Molecular Medicine, Zhongshan School of Medicine, Sun Yat-Sen University, Guangzhou, China; 4 Medical Research Center, Sun Yat-Sen Memorial Hospital, Sun Yat-Sen University, Guangzhou, China; 5 Nanhai Translational Innovation Center of Precision Immunology, Sun Yat-Sen Memorial Hospital, Foshan, China

**Keywords:** Breast cancer, Cancer therapeutic resistance, Cancer metabolism

## Abstract

Aromatase inhibition is an efficient endocrine therapy to block ectopic estrogen production for postmenopausal estrogen receptor (ER)-positive breast cancer patients, but many develop resistance. Here, we show that aromatase inhibitor (AI)-resistant breast tumors display features of enhanced aerobic glycolysis with upregulation of long noncoding RNA (lncRNA) DIO3OS, which correlates with poor prognosis of breast cancer patients on AI therapies. Long-term estrogen deprivation induces DIO3OS expression in ER-positive breast tumor cells, which further enhances aerobic glycolysis and promotes estrogen-independent cell proliferation in vitro and in vivo. Mechanistically, DIO3OS interacts with polypyrimidine tract binding protein 1 (PTBP1) and stabilizes the mRNA of lactate dehydrogenase A (LDHA) by protecting the integrity of its 3’UTR, and subsequently upregulates LDHA expression and activates glycolytic metabolism in AI-resistant breast cancer cells. Our findings highlight the role of lncRNA in regulating the key enzyme of glycolytic metabolism in response to endocrine therapies and the potential of targeting DIO3OS to reverse AI resistance in ER-positive breast cancer.

## Abstract (web summary)

While aromatase inhibitors (AI) are an effective treatment for patients with estrogen receptor positive breast cancer, resistance presents a major obstacle. Here, the authors identify DIO3OS, a long noncoding RNA, as a driver of AI-resistance in breast cancer through the enhancement of aerobic glycolysis.

## Introduction

Aerobic glycolysis, also known as the Warburg effect, is the most striking feature of energy metabolism remodeling in cancers. Tumor cells prefer glycolytic pathways to oxidative phosphorylation, which converts glucose mainly to lactate. Besides, glycolytic metabolites provide necessary materials for biosynthesis, conferring tumor cells with growth advantage^1,2^. Specifically, lactate dehydrogenase A (LDHA) is responsible for converting pyruvate to lactate which has been shown to promote tumor progression by regulating angiogenesis, metastasis, therapeutic resistance as well as chronic inflammation and immune escape^3–6^. Combining glycolytic inhibitors with radiotherapy or chemotherapy can sensitize tumor cells to treatment-induced apoptosis^7–9^, indicating that targeting abnormal glucose metabolism may provide new strategies for reversing therapeutic resistance in cancers.

About two-thirds of breast cancers express estrogen receptor-alpha (ERα, hereinafter referred to as ER), which need endocrine therapy to inhibit estrogen signaling-dependent tumor growth^10,11^. Aromatase inhibitors (AIs) can inactivate the aromatase, a rate-limiting enzyme that converts androgen to estrogen, therefore reducing the estrogen level and suppressing ER-positive breast cancer cell proliferation. In postmenopausal patients, the third-generation AI drugs (letrozole, anastrozole, and exemestane) have become first-line adjuvant therapy for ER-positive breast tumors with therapeutic benefits superior to tamoxifen, once a classical endocrine drug for breast cancer^12–14^. However, acquired resistance to aromatase inhibition accounts for the majority of failure in adjuvant endocrine therapy, making it vital and urgent to decipher the molecular mechanism of AI resistance in ER-positive breast cancer. In current studies, most of the findings focus on the dysregulation of ER and other growth factor receptor signaling, while metabolic reprogramming, one of the key hallmarks of malignant tumors, is rarely discussed in the acquisition of AI resistance^15–17^.

Long noncoding RNAs (LncRNAs) are transcripts of over 200 nucleotides in length, lacking protein-coding capacity. To date, numerous studies have unraveled lncRNAs as pivotal molecules regulating gene expression at the transcriptional, post-transcriptional and epigenetic levels, thereby affecting multiple steps of tumor development^18–20^. Specifically, lncRNA lincRNA-p21^21^, LINK-A^22^, and GLCC1^23^ have been reported to modulate aerobic glycolysis in cancer cells. Previously, we have shown that an exosomal lncRNA HISLA can enhance aerobic glycolysis and induce chemoresistance in breast cancer cells^6^. Despite that, the role of lncRNAs in glycolytic alteration of AI-resistant ER-positive breast cancer and the underlying mechanisms need further elucidation.

In this work, we compare lncRNA expression profiles in the clinical samples of AI-treated ER-positive breast cancer patients and identify DIO3OS as an important RNA regulator in reprograming glucose metabolism in AI-resistant breast tumors.

## Results

### AI-resistant breast cancer exhibits enhanced aerobic glycolysis

**Fig. 1:**
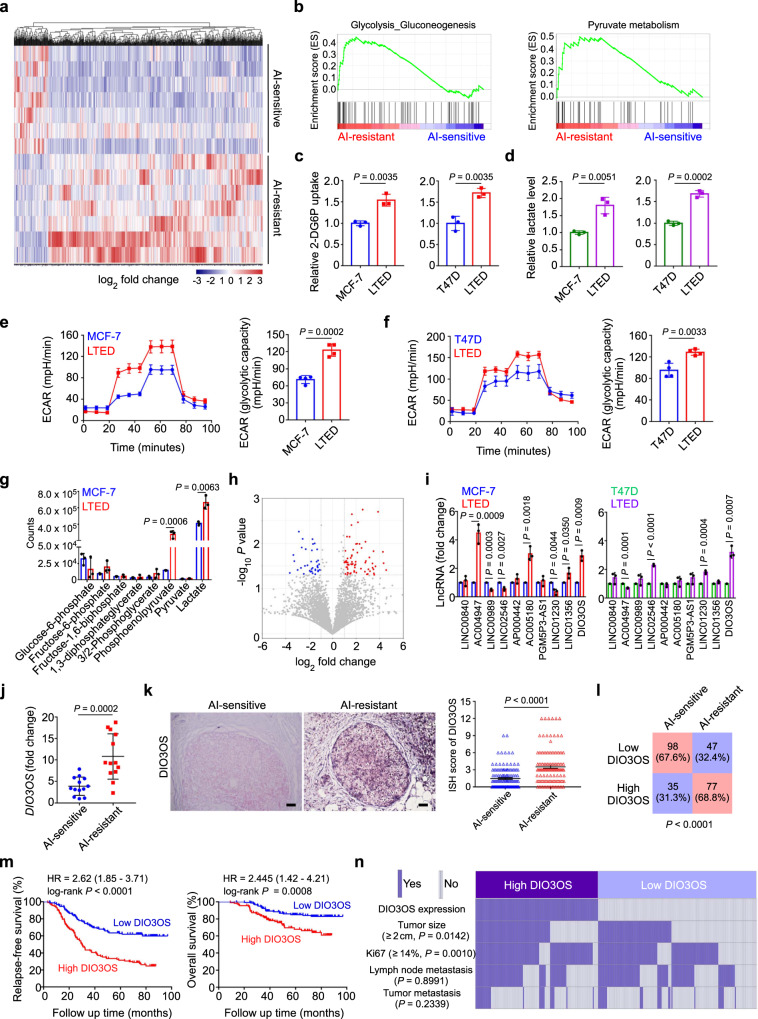
AI-resistant ER-positive breast cancer exhibits enhanced aerobic glycolysis with lncRNA DIO3OS upregulation. **a** Heatmap of expression profile sequencing for breast cancer tissues obtained from patients that were either sensitive or resistant to AI treatment. *n* = 7 biological replicates. **b** GSEA results of the differential gene expression profiles between AI-resistant and AI-sensitive breast cancer. **c**, **d** Glucose uptake (**c**) and lactate production (**d**) of MCF-7/T47D LTED cells in comparison with their parental counterparts. **e**, **f** ECAR values and calculated glycolytic capacity of MCF-7 LTED (**e**) and T47D LTED (**f**) cells compared with their parental counterparts. **g** LC-MS analysis of MCF-7 LTED cells in comparison with parental MCF-7 cells. **h** Volcano plot of lncRNA sequencing data for breast cancer tissues from patients that were either sensitive or resistant to AI treatment. **i** qRT-PCR of the top ten lncRNA candidates in two AI-resistant cell models. **j** qRT-PCR of lncRNA DIO3OS in breast tumor tissues from patients that were sensitive or resistant to AI treatment. Means ± s.d. of *n* = 13 biological replicates are shown. **k** Representative ISH images of DIO3OS in paraffin-embedded breast cancer tissues and the correlation analysis (mean ± s.e.m.) between DIO3OS level and AI treatment response. Tumor tissues were obtained from ER-positive breast cancer patients that were either sensitive (*n* = 133) or resistant (*n* = 124) to AI treatment. Scale bars, 50 μm. **l** Statistical analysis of AI-resistant and AI-sensitive breast tumor tissues under different DIO3OS expression status in cohort. **m** Kaplan–Meier survival curve for relapse-free survival and overall survival in AI-treated ER-positive breast cancer patients with high (*n* = 112) or low (*n* = 145) DIO3OS expression in tumor tissues. HR hazard ratio. **n** Heatmap of the association of the different clinical characters (tumor size, Ki67 index, and metastatic status) with DIO3OS high-expressing and DIO3OS low-expressing AI-treated breast tumors. Means ± s.d. of *n* = 3 (**c**, **d**, **g**, **i**) or *n* = 4 (**e**, **f**) independent experiments yielding similar results are presented, and *P*-values were determined by two-tailed Student’s *t*-test (**c**–**g**, **i**, **j**), Mann–Whitney test (**k**), *χ*2 test (**l**, **n**), or log-rank test (**m**). Source data are provided as a Source Data file.

To explore the molecular features of AI-resistant tumors, we performed high-throughput RNA sequencing to examine the transcription profiles of ER-positive breast cancer tissues from patients with adjuvant AI treatment (Fig. 1a). Gene set enrichment analysis (GSEA) showed that the glycolytic pathway was positively enriched in AI-resistant tissues, compared with the AI-sensitive group (Fig. 1b and Supplementary Fig. 1a, b), suggesting the glucose metabolism reprogramming after AI treatment.

Long-term estrogen deprivation (LTED) has been widely used to mimic clinical AI resistance in ER-positive breast cancer cell^24^. To investigate whether metabolic changes occur in AI-resistant ER-positive breast cancer cells, we examined the uptake of glucose and production of extracellular lactate, a crucial metabolite of glycolysis, in two cell models MCF-7 LTED and T47D LTED, which were insensitive to either estrogen deprivation or AI drugs such as letrozole and anastrozole (Supplementary Fig. 1c, d). We found that both glucose uptake and lactate production were markedly upregulated in LTED cells, which were cultured in estrogen-deprived media for long period (at least 10 months), comparing to parental control cells, which were cultured in estrogen-deprived media for short term (7 days) (Fig. 1c, d). Additionally, glycolytic flux measured by extracellular acidification rates (ECARs) demonstrated that the overall glycolytic flux and glycolytic capacity were enhanced in LTED cells in comparison with parental MCF-7 and T47D cells (Fig. 1e, f).

To further identify the differential metabolites between parental and LTED MCF-7 cells, we screened glycolytic metabolites using liquid chromatography-mass spectrometry (LC-MS). Phosphoenolpyruvate and lactate were significantly upregulated in LTED cells, as compared to parental MCF-7 cells (Fig. 1g). To investigate other potential metabolic alterations in AI-resistance cells, we examined the situations of oxidative phosphorylation and tricarboxylic acid (TCA) cycle in MCF-7 and T47D cells upon estrogen deprivation. However, neither the concentrations of acetyl-CoA nor the oxygen consumption rate (OCR) showed significant difference between the parental and LTED MCF-7/T47D cells (Supplementary Fig. 1e–g). Hence, LTED induces enhanced aerobic glycolysis, rather than oxidative phosphorylation or TCA cycle, in ER-positive breast cancer cells.

Considering the crucial roles of ER signaling in endocrine resistance, we interrogated whether these glycolytic changes in LTED cells were linked to ER expression and activation. Strikingly, parental MCF-7 cells expressed much more ER messenger RNA (mRNA) and protein than the LTED cells did, as revealed by real-time quantitative PCR (qRT-PCR) and immunofluorescence (IF), respectively (Supplementary Fig. 1h, i). Western blot assay further confirmed that both total ER and phosphorylated ER were consistently downregulated in MCF-7 LTED cells in comparison with their parental counterparts (Supplementary Fig. 1j). Meanwhile, a markedly lower basal transcriptional level of ER in MCF-7 LTED cells was determined using the luciferase reporter assay (Supplementary Fig. 1k). To further determine whether ER loss may increase cellular glycolysis following LTED, specific siRNAs and fulvestrant, a well-known ER antagonist, were employed to suppress ER expression in MCF-7 LTED cells, and ER expression was detected by qRT-PCR (Supplementary Fig. 1l) and Western blot (Supplementary Fig. 1m). Subsequent functional assays demonstrated that neither siRNA- nor fulvestrant-mediated ER downregulation in these cells affected the lactate production (Supplementary Fig. 1n) or glycolytic capacity measured by ECARs (Supplementary Fig. 1o). Together, our data suggested that LTED in ER-positive breast cancer cells induces an ER-independent increase in aerobic glycolysis.

### LncRNA DIO3OS is upregulated in AI-resistant breast cancer and clinically associated with AI resistance

To identify the potential regulator driving the glycolytic changes induced by AI resistance, we initially analyzed the mRNA expression profiles and found that the differentially expressed coding genes in the glycolytic pathway were not top listed. Therefore, we focused on the differentially expressed lncRNAs in the top listed panel (Fig. 1h), most of which had unknown biological functions. A total of 60 differentially expressed lncRNA genes were identified between AI-sensitive or -resistant tumor tissues, among which 33 lncRNAs were significantly upregulated, whereas 27 lncRNAs were downregulated in the AI-resistant group (Supplementary Table 1). To narrow down these potential regulators, we performed qRT-PCR to examine the expression level of the top ten upregulated lncRNAs in MCF-7 LTED and T47D LTED cells. DIO3OS was the only concordantly upregulated lncRNA in both LTED cells, with an approximately three-fold increase in comparison to their parental cells (Fig. 1i). Moreover, among the four differentially expressed lncRNAs with statistical significance in cell models, only DIO3OS was markedly increased in the AI-resistant breast cancer tissues compared with those without clinical relapse (Fig. 1j and Supplementary Fig. 2a). Besides, Northern blot confirmed the higher expression of DIO3OS in LTED cells, comparing to their parental counterparts (Supplementary Fig. 2b).

LncRNA DIO3OS is located at human chromosome 14 and transcribed in opposite directions from the DIO3 gene locus. Using 5’- and 3’- rapid amplification of cDNA ends (RACE) and Sanger sequencing, we identified DIO3OS as a 3128 nt intronless transcript (Ensembl/GENCODE transcript ID: ENST00000554735) (Supplementary Fig. 2c, d). To elucidate whether this DIO3OS transcript possesses protein-coding potential, we performed epitope tagging of green fluorescent protein (GFP)-fused putative protein assay and confirmed that DIO3OS-GFP fusion did not encode any protein product (Supplementary Fig. 2e). To detect the subcellular localization of DIO3OS, we extracted the nuclear and cytoplasmic RNA fractions in parental and LTED MCF-7 cells. Subsequent qRT-PCR demonstrated that most DIO3OS was located in the nucleus, while a small portion of DIO3OS was distributed in the cytoplasm (Supplementary Fig. 2f). This finding was also validated by fluorescence in situ hybridization (FISH) of DIO3OS (Supplementary Fig. 2g), implying that DIO3OS might exert its biological function in the nucleus.

To further evaluate the clinical and pathological significance of DIO3OS, we collected 257 paraffin-embedded tumor tissue samples from ER-positive breast cancer patients who received postoperative AI treatment. In situ hybridization (ISH) assay showed that DIO3OS expression was significantly higher in tumor tissues from patients who developed AI resistance than in those without clinical relapse (*P* < 0.0001, Fig. 1k, l). More importantly, high DIO3OS level was associated with poor relapse-free survival (*P* < 0.0001) and overall survival (*P* = 0.0008) of these patients (Fig. 1m). Moreover, DIO3OS expression positively correlated with both tumor size (*P* = 0.0142) and Ki67 index (*P* = 0.0010), but not with lymph node or distant metastasis (Fig. 1n and Supplementary Table 2). Collectively, these data indicated that DIO3OS is highly expressed in AI-resistant tumors and clinically relevant to breast cancer resistance to AI treatment.

### DIO3OS regulates the proliferation and aerobic glycolysis of LTED cells

**Fig. 2:**
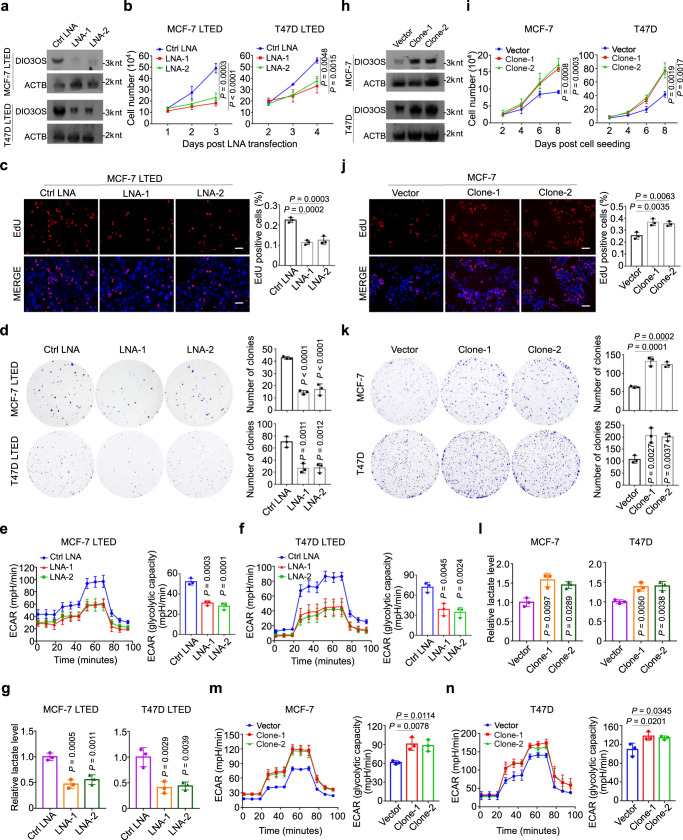
DIO3OS regulates the proliferation and aerobic glycolysis of LTED cells. **a** Northern blot of DIO3OS-knockdown efficiency in MCF-7/T47D LTED cells by the indicated LNAs. ACTB serves as loading control RNA. nt, nucleotide. **b** Growth curves of MCF-7/T47D LTED cells transfected with control or DIO3OS-targeting LNAs. **c** Representative immunofluorescence images and quantification of EdU-incorporated MCF-7 LTED cells transfected with control or DIO3OS-targeting LNAs. Scale bars, 50 μm. **d** Representative images and quantification of plate clone formation of MCF-7/T47D LTED cells transfected with control or DIO3OS-targeting LNAs. **e**, **f** ECAR values and calculated glycolytic capacity of MCF-7 LTED (**e**) and T47D LTED (**f**) transfected with control or DIO3OS-targeting LNAs. **g** Lactate production of MCF-7/T47D LTED cells transfected with control or DIO3OS-targeting LNAs. **h** Northern blot of DIO3OS overexpression efficiency in parental MCF-7/T47D cells. ACTB serves as loading control RNA. nt, nucleotide. **i** Growth curves of parental MCF-7/T47D cells transfected with control or DIO3OS overexpression plasmids. **j** Representative immunofluorescence images and quantification of EdU-incorporated parental MCF-7 cells transfected with control or DIO3OS overexpression plasmids. Scale bars, 50 μm. **k** Representative images and quantification of plate clone formation of parental MCF-7/T47D cells transfected with control or DIO3OS overexpression plasmids. **l** Lactate production of parental MCF-7/T47D cells transfected with control or DIO3OS overexpression plasmids. **m**, **n** ECAR values and calculated glycolytic capacity of parental MCF-7 (**m**) and T47D (**n**) cells transfected with control or DIO3OS overexpression plasmids. Means ± s.d. of experimental triplicates (**b**–**g**, **i**–**n**), one representative experiment out of three that were similar (**a**, **h**) are shown, and *P*-values were assessed with two-sided one-way ANOVA with Dunnett’s multiple-comparisons test. Source data are provided as a Source Data file.

To investigate the functional role of DIO3OS in AI resistance, we performed loss-/gain-of-function assays. Due to the nuclear localization of DIO3OS, we employed locked nucleic acids (LNAs), instead of siRNAs, to silenced its expression^25^.With the effectiveness confirmed by qRT-PCR (Supplementary Fig. 3a) and Northern blot (Fig. 2a), two LNAs were applied for further knockdown experiments. As revealed by cell counting and MTT assays, silencing DIO3OS significantly impaired the growth of MCF-7 LTED and T47D LTED cells (Fig. 2b and Supplementary Fig. 3b). More specifically, DIO3OS knockdown in LTED cells reduced the proportion of proliferating cells, as evidenced by EdU incorporation assay (Fig. 2c and Supplementary Fig. 3c) as well as Ki67 staining (Supplementary Fig. 3d). In parallel, the clonogenicity of LTED cells were decreased upon DIO3OS silencing in the colony formation assay (Fig. 2d). It was worth noting that LNA-mediated DIO3OS knockdown (Supplementary Fig. 3e) hardly affected the growth and proliferation of parental MCF-7 cells, as detected by cell counting, MTT, EdU incorporation and clone formation assays (Supplementary Fig. 3f–i). As DIO3OS expression was extremely low in parental T47D cells, we speculated that downregulating DIO3OS would not affect the biological behavior of T47D cells.

Given that aerobic glycolysis was enhanced in AI-resistant breast cancer, we next evaluated the effect of silencing DIO3OS on the glycolytic metabolism in these cells. Indeed, knocking down DIO3OS dramatically reduced the ECAR level as well as glycolytic capacity in MCF-7 LTED and T47D LTED cells, as compared to their control cells (Fig. 2e, f). In addition, glucose consumption assay and lactate production assay accordantly validated that DIO3OS silencing in LTED cells significantly impaired their glucose uptake capacity (Supplementary Fig. 3j) and lactate levels (Fig. 2g).

Moreover, we determined the contribution of DIO3OS in tamoxifen-resistant model as well as HER2-positive and triple-negative breast cancer (TNBC). Unlike AI-resistant models, DIO3OS expression decreased in tamoxifen-resistant MCF-7 cells compared to their parental line (Supplementary Fig. 3k). Among the TNBC lines, only BT549 cells exhibited moderate elevation of DIO3OS expression, by twofold higher than MCF-7 cells, but the lncRNA was almost not detected in MDA-MB-231, MDA-MB-468, Hs578T, and MDA-MB-436 cells (Supplementary Fig. 3l). As for HER2-positive breast cancer cells, except that SKBR3 revealed similar DIO3OS expression as MCF-7, both BT474 and MDA-MB-361 cells expressed lower level of DIO3OS (Supplementary Fig. 3l). Since BT549 cells and SKBR3 cells exhibited moderate DIO3OS expression, we then explored the potential function of DIO3OS in these two cell lines. Both cell counting and MTT assays revealed that with DIO3OS knockdown by LNAs (Supplementary Fig. 3m), the growth of BT549 cells was slightly impaired, while SKBR3 cells remained largely unaffected (Supplementary Fig. 3n, o), indicating a less important role of DIO3OS in these ER-negative or HER2-positive breast cancer.

In the gain-of-function assays, we transfected DIO3OS-expressing pcDNA3.1 plasmid into parental MCF-7 and T47D cells, followed by G418 selection, to establish stable DIO3OS-overexpressing cells (Fig. 2h and Supplementary Fig. 3p). Under estrogen deprivation condition, DIO3OS-overexpressing MCF-7 and T47D cells grew faster than those transfected with control vectors (Fig. 2i). EdU incorporation assay further demonstrated that DIO3OS overexpression supported the estrogen-independent proliferation of parental MCF-7 and T47D cells (Fig. 2j and Supplementary Fig. 3q). Also, DIO3OS-overexpressing MCF-7 and T47D cells featured enhanced colony-forming capacity in estrogen-deprived context (Fig. 2k). Moreover, enforced DIO3OS expression in parental MCF-7 and T47D cells promoted their glucose metabolism, as featured by higher glucose uptake (Supplementary Fig. 3r), lactate production (Fig. 2l) as well as ECAR levels (Fig. 2m, n). Together, these data demonstrated that DIO3OS promotes ER-positive breast tumor cell proliferation and aerobic glycolysis in an estrogen-independent manner.

### DIO3OS interacts with PTBP1 in the nucleus

**Fig. 3:**
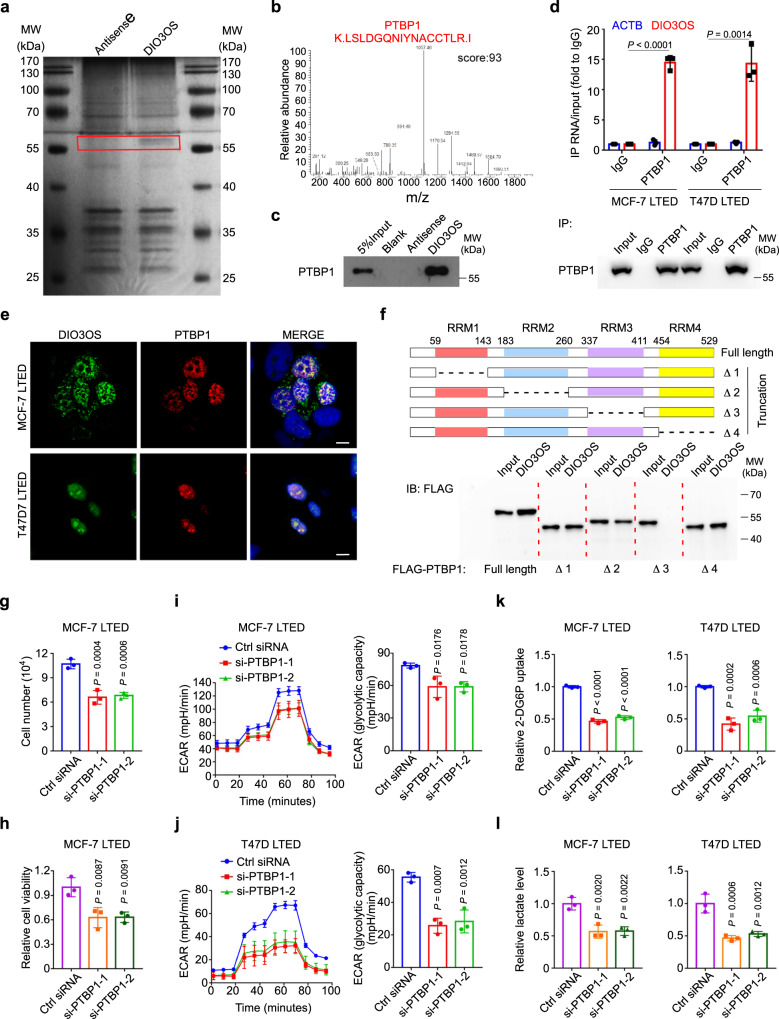
DIO3OS interacts with PTBP1 in the nucleus. **a** Biotin-RNA pulldown assay using full-length DIO3OS transcript (sense) and the antisense RNA, followed by silver staining. Red box indicates the differential band. MW molecular weight. **b** Mass spectrometry profile of DIO3OS-binding protein PTBP1 with high score. **c** Biotin-RNA pulldown assay followed by western blot demonstrating the interaction of DIO3OS and PTBP1. MW, molecular weight. **d** RNA immunoprecipitation assay with the anti-PTBP1 antibody demonstrating the interaction of DIO3OS and PTBP1 in MCF-7/T47D LTED cells. MW, molecular weight. **e** Fluorescence assessment of the subcellular colocalization of DIO3OS and PTBP1 in MCF-7/T47D LTED cells. DIO3OS and PTBP1 were detected respectively by RNA FISH (green) and immunofluorescence (red) staining. Scale bars, 10 μm. **f** Biotin-RNA pulldown assay followed by western blot demonstrating the interaction of DIO3OS and the RNA recognition motifs (RRMs) of PTBP1. Schematic diagram for PTBP1 truncation variants deleting the RRM1, RRM2, RRM3, or RRM4 (upper). Representative western blot for in vitro binding of FLAG-tagged PTBP1 truncation variants with DIO3OS (bottom). MW, molecular weight. **g**, **h** Cell number counting (**g**) and MTT assay (**h**) of MCF-7 LTED cells transiently transfected with control or PTBP1 siRNAs. **i**, **j** ECAR values and calculated glycolytic capacity of MCF-7 LTED cells (**i**) and T47D LTED cells (**j**) transiently transfected with control or PTBP1 siRNAs. **k** Glucose uptake of MCF-7/T47D LTED cells transiently transfected with control or PTBP1 siRNAs. **l** Lactate production of MCF-7/T47D LTED cells transiently transfected with control or PTBP1 siRNAs. Means ± s.d. of experimental triplicates (**d**, **g**–**l**), one representative experiment out of three that were similar (**a**–**c**, **e**, **f**) are shown, and *P-*values were determined using two-tailed Student’s *t*-test (**d**), two-sided one-way ANOVA with Dunnett’s multiple-comparisons test (**g**–**l**). Source data are provided as a Source Data file.

To dissect the molecular mechanism of DIO3OS in cancer metabolism, we applied RNA pulldown assay with an in vitro transcribed biotin-labeled DIO3OS RNA to screen DIO3OS-interacting proteins. A specific band between 55–70 kDa pulled down by DIO3OS, but not the antisense control RNA, was sent to mass spectrometric analysis (Fig. 3a). Among all the RNA pulldown proteins, polypyrimidine tract binding protein 1 (PTBP1), a nuclear alternative splicing regulator, was the most enriched one with a highest protein sequence coverage (Fig. 3b, Supplementary Tables 3–4, Supplementary Data 1 and Supplementary Note 1). Western blot further confirmed that PTBP1 bound specifically to DIO3OS (Fig. 3c). Also, RNA immunoprecipitation (RNA-IP) assay using an anti-PTBP1 antibody was performed, which showed that DIO3OS was retrieved with ~15-fold enrichment in the anti-PTBP1 immunoprecipitates, compared with control IP reactions using IgG in LTED cells (Fig. 3d). To exclude indirect interactions of DIO3OS and PTBP1, we further performed crosslinking immunoprecipitation (CLIP) assay in LTED cells by using formaldehyde to stabilize the physiological interactions of RNA-protein complexes. DIO3OS was enriched more than 20-fold in the anti-PTBP1 immunoprecipitates comparing to the IgG control (Supplementary Fig. 4a). Thus, an enhanced retrieval of DIO3OS was obtained in CLIP assay, relative to RNA-IP assay. In accordance with the nuclear localization of DIO3OS, confocal microscopy for FISH and immunofluorescence staining demonstrated the colocalization of DIO3OS and PTBP1 in the nucleus of MCF-7 LTED and T47D LTED cells (Fig. 3e). Given that PTBP1 is a hnRNP protein (hnRNP I) containing four RNA recognition motifs (RRMs) connected by three linkers, its specific binding site with DIO3OS was further identified by using full-length and truncated PTBP1 constructs tagged with FLAG (Fig. 3f, upper). RNA pulldown assays demonstrated that the RRM3 of PTBP1 protein, rather than other RRM-type RNA-binding motifs, specifically interacted with the in vitro transcribed DIO3OS RNA (Fig. 3f, bottom). In consistence with previous reports^26–28^, RRM3 is a recognized binding domain that contributes to RNA-binding specificity of PTBP1. Correspondingly, the PTBP1-targeting pyrimidine-rich motifs (e.g., UCUU, UCUUC, UCUCU, UUCUCU, CUCUCU) were found widespread in the DIO3OS RNA. By comparing the secondary structures of different DIO3OS variants predicted by RNAfold^29^ (Supplementary Fig. 4b–g), we found that 60% PTBP1-binding motifs clustered in the region of nt 850–1930 of ENST00000554735, which only accounted for a third of its length and formed multiple stem-loop structures (Supplementary Fig. 4h). These data suggested that DIO3OS specifically binds to PTBP1 in the nucleus of LTED breast cancer cells.

PTBP1 modulates the alternative splicing of many genes under certain contexts, resulting in the production of miscellaneous RNA transcripts with distinct functions^28,30^. To assay the potential regulation of PTBP1 on DIO3OS, we knocked down PTBP1 by two siRNAs (Supplementary Fig. 4i, j) and found that there were no significant changes in DIO3OS expression, as determined by qRT-PCR (Supplementary Fig. 4k) and Northern blot (Supplementary Fig. 4l). Since PTBP1 has been reported to participate in various processes of cancer progression, involving cell proliferation and metabolic reprogramming^31^, we then explored the functional role of PTBP1 in LTED cells. As revealed by cell counting and MTT assays, LTED cell growth was significantly suppressed by siRNA-mediated PTBP1 knockdown (Fig. 3g, h). EdU incorporation assay further substantiated that PTBP1 knockdown inhibited LTED cell proliferation (Supplementary Fig. 4m). We next tested whether silencing PTBP1 influenced the aerobic glycolysis in LTED cells. Consistent with the function of DIO3OS in LTED cells, knocking down PTBP1 decreased the ECAR levels as well as glycolytic capacity in MCF-7 LTED and T47D LTED cells (Fig. 3i, j). Both glucose consumption and lactate production were also impaired in PTBP1-knockdown LETD cells (Fig. 3k, l). Therefore, DIO3OS specifically interacts with nuclear PTBP1, a splicing regulator that supports the growth and aerobic glycolysis of LTED breast cancer cells.

### DIO3OS regulates LTED cell proliferation and glycolysis through PTBP1

**Fig. 4:**
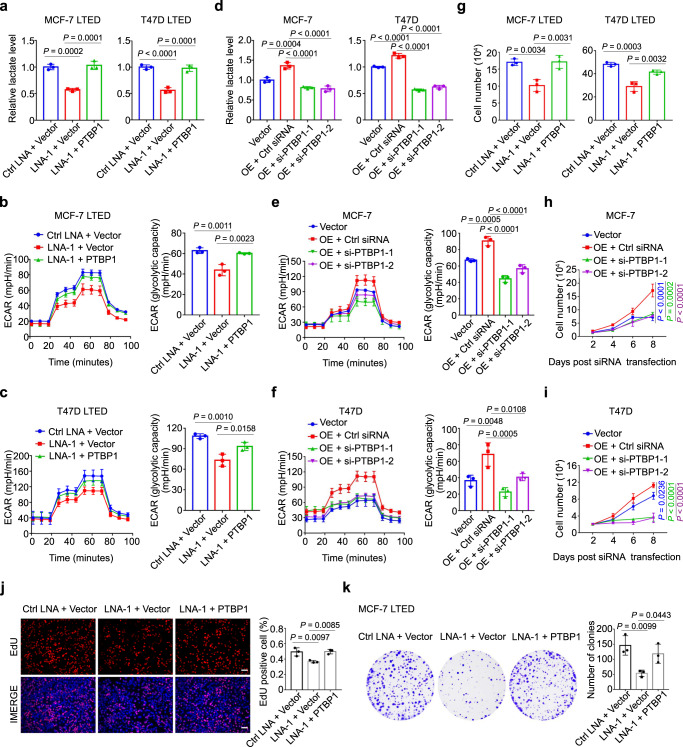
DIO3OS regulates LTED cell proliferation and glycolysis through PTBP1. **a** Lactate production of MCF-7/T47D LTED cells co-transfected with control LNA/DIO3OS-targeting LNA and control vector/PTBP1 overexpression plasmid. **b**, **c** ECAR values and calculated glycolytic capacity of MCF-7 LTED (**b**) and T47D LTED (**c**) cells with the indicated treatments. **d** Lactate production of parental MCF-7/T47D cells transfected with control construct or co-transfected with DIO3OS expression plasmid and PTBP1 siRNAs or control siRNA. **e**, **f** ECAR values and calculated glycolytic capacity of parental MCF-7 (**e**) and T47D (**f**) cells with the indicated treatments. **g**–**i** Cell number counting of MCF-7/T47D LTED cells (**g**) and parental MCF-7/T47D cells (**h**, **i**) with the indicated treatments. **j** Representative immunofluorescence images and quantification of EdU-incorporated MCF-7 LTED cells with the indicated treatments. Scale bars, 50 μm. **k** Representative images and quantification of plate clone formation of MCF-7 LTED cells with the indicated treatments. Means ± s.d. of experimental triplicates (**a**–**k**) are shown, and all *P*-values were analyzed using two-sided one-way ANOVA with Dunnett’s multiple-comparisons test. Source data are provided as a Source Data file.

To validate that DIO3OS exerts its function via direct interaction with PTBP1, we conducted a series of rescue experiments. First, we transfected MCF-7/T47D LTED cells with control LNA or DIO3OS LNA, along with control vector, full-length PTBP1 construct or PTBP1 truncation variant deleting the RRM3, and then measured their lactate production as well as ECAR levels. Compared with control LNA/vector-transfected MCF-7/T47D LETD cells, co-transfection with DIO3OS LNA and full-length PTBP1 construct rescued the lactate production that was impaired in cells with DIO3OS downregulation and without PTBP1 overexpression (Fig. 4a), while co-transfection of DIO3OS LNA and PTBP1 RRM3-deleted construct resembled the result of DIO3OS knockdown (Supplementary Fig. 5a). Similar results were obtained from the glycolytic seahorse assay (Fig. 4b, c and Supplementary Fig. 5b). On the other hand, enhanced lactate production and ECAR levels in DIO3OS-overexpressing parental MCF-7 cells were inhibited by PTBP1 siRNAs, comparing to control siRNAs (Figs. 4d, left, and 4e). These results were also readily detected in T47D cells (Figs. 4d, right, and 4f).

In the cell growth experiments, comparing to control sets, DIO3OS LNA transfection led to decreased cell number in both MCF-7 LTED and T47D LTED cells, whereas PTBP1 overexpression rescued cell growth almost back to the control levels (Fig. 4g). Specifically, MTT assay showed that contrary to the full-length PTBP1, PTBP1 RRM3-deleted construct failed to rescue the cell viability that were dampened in cells co-transfected with DIO3OS LNA (Supplementary Fig. 5c). In parallel, PTBP1 siRNAs abolished the stable DIO3OS overexpression-increased cell growth in parental MCF-7 and T47D cells (Fig. 4h, i). Similar result of cell proliferation was obtained from the EdU incorporation assay (Fig. 4j and Supplementary Fig. 5d–f). Moreover, PTBP1 upregulation rescued the colony-forming capacity of LTED cells with DIO3OS knockdown (Fig. 4k and Supplementary Fig. 5g), and vice versa (Supplementary Fig. 5h). Together, DIO3OS regulates the aerobic glycolysis as well as proliferation in LTED breast cancer cells via PTBP1.

### DIO3OS interacts with PTBP1 to upregulate LDHA mRNA stability

**Fig. 5:**
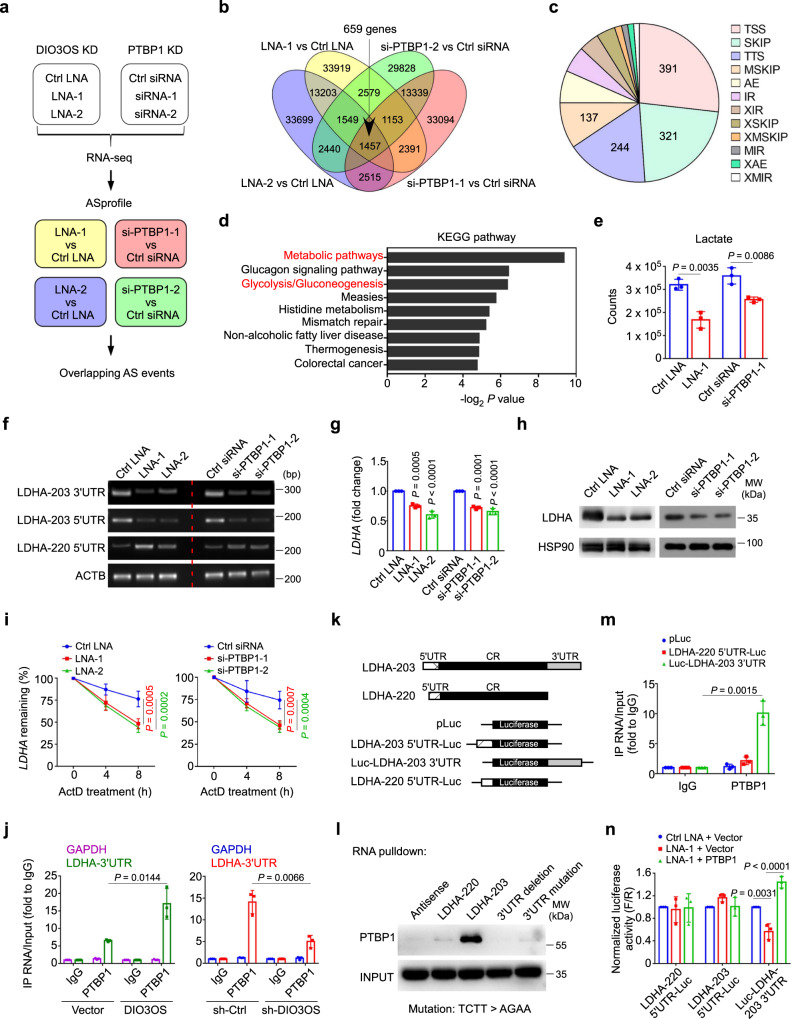
DIO3OS interacts with PTBP1 to upregulate LDHA mRNA stability. **a** Schematic diagram of alternative splicing (AS) analysis for RNA-seq of MCF-7 LTED cells with or without DIO3OS/PTBP1 knockdown (KD). **b** Venn diagram of the differentially spliced events in MCF-7 LTED cells transfected with control LNA/siRNA and DIO3OS LNA or PTBP1 siRNA. **c** Pie chart of twelve types of the differentially splicing events occurred in DIO3OS/PTBP1-knockdown MCF-7 LTED cells. **d** KEGG pathway enrichment of the differentially spliced genes in DIO3OS/PTBP1-knockdown MCF-7 LTED cells using the DAVID bioinformatics tool are plotted as the −log_2_ *P-*value. **e** LC-MS analysis of lactate production in MCF-7 LTED cells with indicated treatment. **f** PCR and agarose gel of the 5’/3’UTR of LDHA mRNA in MCF-7 LTED cells with indicated treatment. **g** qRT-PCR of LDHA mRNA in MCF-7 LTED cells with indicated treatment. **h** Immunoblot of LDHA expression in MCF-7 LTED cells with indicated treatment. MW molecular weight. **i** Half-life of LDHA mRNA in MCF-7 LTED cells with indicated treatment was determined by using 10 µg/mL actinomycin D (ActD) at indicated time points. **j** RNA immunoprecipitation assay with the anti-PTBP1 antibody revealing the interaction of PTBP1 and LDHA variant harboring 3’UTR in parental and LTED MCF-7 cells with indicated treatments. **k** Schematic diagram of the full length of two different LDHA variant-expressing plasmids, and the luciferase reporter plasmids fused with different 5’/3’UTR sequences of LDHA. **l** Biotin-RNA pulldown followed by western blot demonstrating the interaction of PTBP1 and different LDHA variants with or without intact 3’UTR. MW molecular weight. **m** RNA immunoprecipitation assay with the anti-PTBP1 antibody revealing the interaction of PTBP1 and LDHA 5’/3’UTR in MCF-7 LTED cells transfected with different LDHA-UTR-fused luciferase reporter plasmids. **n** Luciferase reporter assay in MCF-7 LTED with indicated treatment. Means ± s.d. of *n* = 3 (**e**, **g**, **j**, **m**, **n**) or *n* = 4 (**i**) independent experiments yielding similar results, one representative experiment out of three that were similar (**f**, **h**, **l**) are shown, and *P-*values were analyzed using two-tailed Student’s *t*-test (**e**, **j**, **m**), two-sided one-way ANOVA with Dunnett’s multiple-comparisons test (**g**, **i**, **n**). Source data are provided as a Source Data file.

To explore how DIO3OS functions through interacting with PTBP1, we employed two sets of high-throughput sequencing to compare the transcription profiles of MCF-7 LTED cells with and without LNA-mediated DIO3OS knockdown, as well as siRNA-mediated PTBP1 knockdown, respectively (Fig. 5a). Given that PTBP1 was a suppressor of alternative splicing (AS)^32^ and DIO3OS potentially exerted an inhibitory effect on gene splicing by binding with PTBP1, we focused on the splicing events concordantly increased in DIO3OS and PTBP1-knockdown cells. Twelve basic types of AS patterns in each sample were analyzed with ASprofile program and the differential AS events were identified in four sets of samples (Knockdown vs Control) (Fig. 5a and Supplementary Data 2). By overlapping all four sets of the differential AS events, we identified 1457 events in 659 genes from concurrent DIO3OS and PTBP1-knockdown cells relative to control (Fig. 5b and Supplementary Data 2). Among these events, AS occurring at transcription start site (TSS) was the predominant type (27%), followed by skipped exon (SKIP) (22%) and transcription terminal site (TTS) (17%) (Fig. 5c). The remaining types of AS events, including multiple skipped exon (MSKIP), alternative exon ends (AE), intron retention (IR), approximate intron retention (XIR), approximate skipped exon (XSKIP), approximate multiple skipped exon (XMSKIP), multiple intron retention (MIR), approximate alternative exon ends (XAE), and approximate multiple intron retention (XMIR), occurred at lower frequencies (Fig. 5c). With the DAVID Functional Annotation Tool^33^, KEGG pathway analysis revealed that many of differentially spliced genes were clustered in metabolic pathways, especially the glycolysis/gluconeogenesis pathway (Fig. 5d). These genes included LDHA, ALDH3A1, ALDOC, PFKP, ENO3, ALDH3B2, and PCK2. As such, our data suggested that DIO3OS and PTBP1 might regulate the alternative splicing of certain key glycolysis-related genes.

To narrow down the candidate genes, LC-MS was performed to screen the glycolytic metabolites affected by DIO3OS and PTBP1. We found both D-glucose 6-phosphate (G6P) and lactate were significantly decreased upon DIO3OS and PTBP1 knockdown (Fig. 5e and Supplementary Fig. 6a). Hexokinase-2 (HK2) and lactate dehydrogenase A (LDHA) are the two crucial metabolic enzymes that catalyze the production of G6P and lactate, respectively. Backtracking the alternatively spliced genes in DIO3OS and PTBP1-knockdown cells, besides LDHA, ALDH3A1, ALDOC, PFKP, and ENO3 also affect lactate production. Therefore, we used RT-PCR to examine the splicing events of these genes upon DIO3OS and PTBP1 knockdown, and found that AS events occurred only in the mRNA of LDHA (Fig. 5f), but not other glycolytic enzymes.

LDHA is the key enzyme which can catalyze the conversion of pyruvate into lactate, a crucial process of glycolysis. According to the ISOexpresso database, the most common LDHA transcript in breast cancer cells is LDHA-203, which harbors a 357-nt 5’ untranslated region (UTR) as well as a 567-nt 3’UTR. The LDHA isoform encoded by this transcript has also been chosen as the canonical sequence, as shown in UniProtKB database. The AS event in LDHA mRNA from DIO3OS and PTBP1-knockdown cells was identified as TSS by the ASprofile software, which occurred at chr11:18,396,554–18,396,968, pointing to another transcript variant LDHA-220. As revealed by the UCSC Genome Browser, LDHA-220 not only harbors a 5’UTR differing from that of LDHA-203, but also features a complete deletion of 3’UTR (Supplementary Fig. 6b). To confirm these alterations of UTR sequences in LDHA mRNA, specific primers targeting the 3’UTR of LDHA-203, 5’UTRs of LDHA-203 and LDHA-220 were designed and subjected to RT-PCR assay (Supplementary Fig. 6b). The products were further visualized in agarose gels, which showed that both 3’UTR and 5’UTR of LDHA-203 were strikingly reduced while the 5’UTR of LDHA-220 was upregulated in LTED cells with DIO3OS or PTBP1 knockdown (Fig. 5f and Supplementary Fig. 6c), indicating an increase in LDHA-220 transcript featuring 3’UTR deficiency after DIO3OS or PTBP1 downregulation. To further demonstrate the impacts of UTR sequence changes on mRNA and protein expression, qRT-PCR was performed and revealed that only *LDHA*, but not *PFKP*, *ALDOC*, *ENO3*, or *HK2*, exhibited decreased mRNA level in DIO3OS and PTBP1-knockdown LTED cells (Fig. 5g and Supplementary Fig. 6d). And the protein level of LDHA upon DIO3OS or PTBP1 knockdown was consistently reduced, as determined by western blot (Fig. 5h). Thus, we focused on LDHA for further investigation.

To elucidate the potential regulation of LDHA by DIO3OS in BT549 and SKBR3 cells, we also detected the LDHA expression in these DIO3OS-knockdown cells. In consistence with the phenotypic assays, silencing DIO3OS in BT549 and SKBR3 cells did not affect the LDHA mRNA level (Supplementary Fig. 6e). Also, qRT-PCR revealed that both LDHA-203 and LDHA-220 expression remained unchanged in DIO3OS-knockdown BT549 cells and SKBR3 cells (Supplementary Fig. 6f), indicating that DIO3OS might not affect LDHA splicing in these cells.

5’UTR and 3’UTR harbor important regulatory elements for mRNA processing. Particularly, 5’UTR has been reported to regulate the translation initiation^34^ while 3’UTR is more likely to be involved in the mRNA transport and stabilization^35^. To test whether DIO3OS and PTBP1 stabilize LDHA mRNA, we used actinomycin D (ActD) to inhibit mRNA transcription with the proto-oncogene *c-Myc* as an unstable control, whose mRNA level was strikingly decreased 2 h following ActD treatment (Supplementary Fig. 6g). As revealed in Fig. 5i, a significant reduction of the LDHA mRNA stability was detected in MCF-7 LTED cells with either DIO3OS or PTBP1 knockdown. To further dissect the interaction between PTBP1 and LDHA mRNA, as well as the role of DIO3OS therein, we examined whether DIO3OS directly regulated the PTBP1 expression. However, PTBP1 protein level was not changed after DIO3OS knockdown (Supplementary Fig. 6h). Then we performed the RNA-IP assay to detect whether PTBP1 interacted with LDHA mRNA, and whether DIO3OS affected the interaction. In parental MCF-7 cells transfected with control vector, LDHA mRNA harboring 3’UTR was enriched ~5 folds in the anti-PTBP1 immunoprecipitates, compared to the IgG immunoprecipitates, whereas in DIO3OS-overexpressing MCF-7 cells, the retrieval of LDHA mRNA with 3’UTR by anti-PTBP1 antibody was significantly increased to ~15 folds (Fig. 5j, left). These RNA-protein (RNP) complexes were also readily detected in MCF-7 LTED cells, where the anti-PTBP1 immunoprecipitates retrieved much more LDHA mRNA with 3’UTR in sh-Ctrl cells than in DIO3OS-knockdown cells (Fig. 5j, right). These data suggested that DIO3OS promotes the interaction between PTBP1 and LDHA mRNA that harbors 3’UTR.

To address whether PTBP1 directly binds to the 3’UTR within LDHA mRNA, we constructed plasmids for the two LDHA transcripts, LDHA-220 and LDHA-203 (Fig. 5k). RNA pulldown assay followed by Western blot demonstrated that full length of LDHA-203 harboring 3’UTR retrieved a significantly stronger PTBP1 band from total protein lysates than LDHA-220 and antisense did (Fig. 5l). It has been known that PTBP1 binds to RNA through short, single-strand pyrimidine motifs, including UCUU, UCUUC, UCUUU, UCUCU, UUCUCU, and CUCUCU^28,30^. Thus, we searched for these motifs in the 3’UTR of LDHA-203, where six UCUU sequences were identified. We then generated a mutant of LDHA-203 3’UTR by replacing TCTT with AGAA. Western blot following RNA pulldown assay showed that unlike the intact LDHA-203, LDHA-203 with 3’UTR mutation failed to enrich PTBP1, which was similar to 3’UTR-deleted LDHA-203 (Fig. 5l), indicating that UCUU sequences in the 3’UTR accounted for the binding of LDHA-203 to PTBP1. To recapitulate the interaction between PTBP1 and LDHA 3’UTR or 5’UTR ex vivo, LTED cells were transfected with chimeric reporter gene constructs expressing luciferase fused with either LDHA-203 3’UTR or LDHA-220 5’UTR (negative control). The RNP complexes were immunoprecipitated by anti-PTBP1 antibody and qRT-PCR revealed a much more enrichment in the 3’UTR-containing luciferase chimeric transcript than the 5’UTR-lucifrease reporter, substantiating that PTBP1 preferentially bound to the 3’UTR, but not to 5’UTR, of the LDHA mRNA in LTED cells (Fig. 5m).

To further validate that DIO3OS modulates LDHA expression by virtue of PTBP1 binding to LDHA 3’UTR, we transfected reporter plasmids containing luciferase gene along with LDHA-220 5’UTR, LDHA-203 5’UTR, or LDHA-203 3’UTR (Fig. 5k) into MCF-7 LTED cells with or without DIO3OS knockdown or PTBP1 overexpression. Luciferase reporter assay showed that partial silencing of DIO3OS by LNA only caused reduction in the luciferase activity of cells with luc-3’UTR (LDHA-203) expressed, while in the presence of PTBP1-overexpressing plasmid, the luciferase activity in luc-3’UTR (LDHA-203) construct was dramatically restored (Fig. 5n). However, similar phenomena didn’t occur in contexts with constructs harboring 5’UTR (Fig. 5n). Collectively, our findings indicated that DIO3OS promotes the binding of PTBP1 to LDHA 3’UTR, therefore stabilizing LDHA mRNA and resulting in more LDHA expression and lactate production.

### Functional LDHA-203 is upregulated in LTED cells and AI-resistant breast cancer cells

**Fig. 6:**
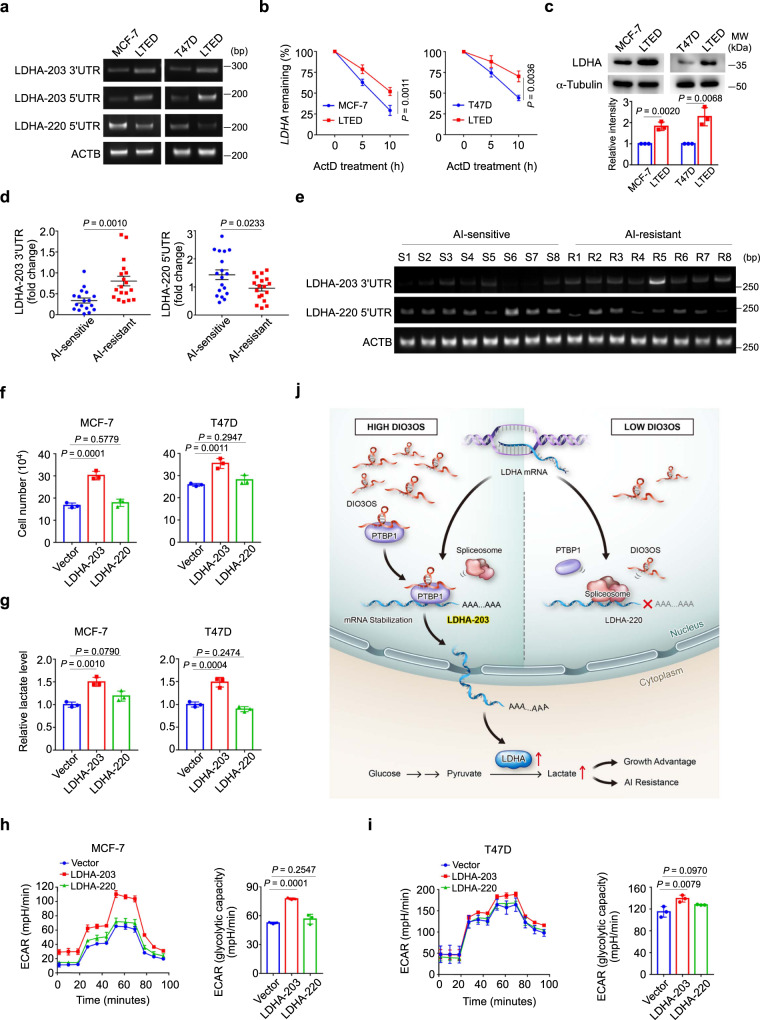
Functional LDHA-203 is upregulated in LTED cells and AI-resistant breast cancer cells. **a** PCR and agarose gel of the 5’/3’UTR of LDHA mRNA in parental MCF-7/T47D cells and their LTED derivatives. **b** Half-life of LDHA mRNA in parental MCF-7/T47D cells and their LTED derivatives was determined by using 10 µg/mL actinomycin D (ActD) at indicated time points. Means ± s.d. of *n* = 4 (MCF-7 cells) or *n* = 3 (T47D cells) independent experiments are shown. **c** Immunoblot and quantification of LDHA protein expression in parental MCF-7/T47D cells and their LTED derivatives. MW molecular weight. **d** qRT-PCR of the 5’/3’UTR of LDHA mRNA in AI-sensitive or AI-resistant breast cancer tissues. Mean ± s.e.m. of *n* = 18 biological replicates are shown. **E** PCR and agarose gel of the 5’/3’UTR of LDHA mRNA in AI-sensitive or AI-resistant breast cancer tissues. *n* = 8 biological replicates. **f**, **g** Cell number counting (**f**) and lactate production (**g**) of parental MCF-7/T47D cells transiently transfected with different LDHA variant-expressing plasmids or control vector. **h**, **i** ECAR values and calculated glycolytic capacity of parental MCF-7/T47D cells with indicated treatments. **j** Graphical illustration of the DIO3OS/PTBP1/LDHA glycolysis cascade acting as a switch regulating the glucose metabolism in breast cancer adaptation to LTED by stabilizing LDHA mRNA. Means ± s.d. of experimental triplicates (**c**, **f**–**i**), one representative experiment out of three that were similar (**a**, **e**) are shown, and *P-*values were analyzed by two-tailed Student’s *t*-test (**b**–**d**), two-sided one-way ANOVA with Dunnett’s multiple-comparisons test (**f**–**i**). Source data are provided as a Source Data file.

Next, we examined whether these alterations of UTR sequences in LDHA mRNA occurred in parental MCF-7 and T47D cells and their LTED derivatives. RT-PCR using specific primers towards LDHA-203 3’UTR, LDHA-203 5’UTR and LDHA-220 5’UTR showed that LDHA-220, harboring the peculiar 5’UTR and lacking 3’UTR, mainly existed in parental MCF-7 and T47D cells, whereas LDHA-203, harboring longer 5’UTR and 3’UTR, significantly increased in LTED cells (Fig. 6a), suggesting that LDHA-220 in parental cells might be replaced by LDHA-203 following LTED treatment. To figure out whether such sequence variations in UTRs affect the stability of LDHA mRNA in parental and LTED cells, we inhibited mRNA transcription through ActD treatment, which was proven to be effective using c-Myc mRNA as a positive control (Supplementary Fig. 6g). Subsequent qRT-PCR revealed an over 10-h half-life of LDHA mRNA in LTED cells, whereas the stability of LDHA mRNA in parental MCF-7 or T47D cells was markedly shortened (Fig. 6b). Meanwhile, LTED cells exhibited a higher LDHA protein level in contrast to their parental cells (Fig. 6c).

Furthermore, we detected the sequence alterations of LDHA mRNA in breast tumor tissues obtained from AI-sensitive and AI-resistant patients using qRT-PCR. In consistence with the result of cell lines, AI-resistant breast cancer samples featured a generally higher expression of LDHA-203 (with 3’UTR) than AI-sensitive groups, while the latter ones exhibited more upregulated expression of LDHA-220 (with unique 5’UTR) (Fig. 6d, e).

To further demonstrate the functionalities of these two LDHA transcripts in ER-positive breast cancer cells, we overexpressed them in parental MCF-7 and T47D cells. Although qRT-PCR with specific primers targeting either 5’UTR of LDHA-203 and LDHA-220 confirmed that both transcript variants were successfully overexpressed 24 h post transfection (Supplementary Fig. 6i), western blot revealed different LDHA protein levels 48 h after ectopic expression of two transcripts (Supplementary Fig. 6j), indicating that LDHA-220 featuring 3’UTR absence might be prone to decay, resulted in lowered protein level than LDHA-203 did. In the phenotypic assays, when compared to the control context, overexpression of LDHA-203, but not LDHA-220, markedly promoted the growth of parental MCF-7 and T47D cells (Fig. 6f). LDHA-203-expressing cells showed enhanced lactate production (Fig. 6g) as well as higher ECAR levels (Fig. 6h, i). Instead, LDHA-220 transfection didn’t enhance the glycolysis of parental MCF-7 and T47D cells, as determined by lactate production and ECAR measurement (Fig. 6g–i). Therefore, LDHA-203, upregulated in AI-resistant breast cancer cells, could increase the lactate production during the glycolytic processes.

Collectively, lncRNA DIO3OS directly interacts with PTBP1 protein in the nucleus and stabilizes the mRNA of LDHA by protecting the integrity of its 3’UTR, which consequently upregulates LDHA expression and activates glycolytic metabolism in AI-resistant breast cancer cells (Fig. 6j).

### DIO3OS promotes ER-positive breast tumor progression in vivo

**Fig. 7:**
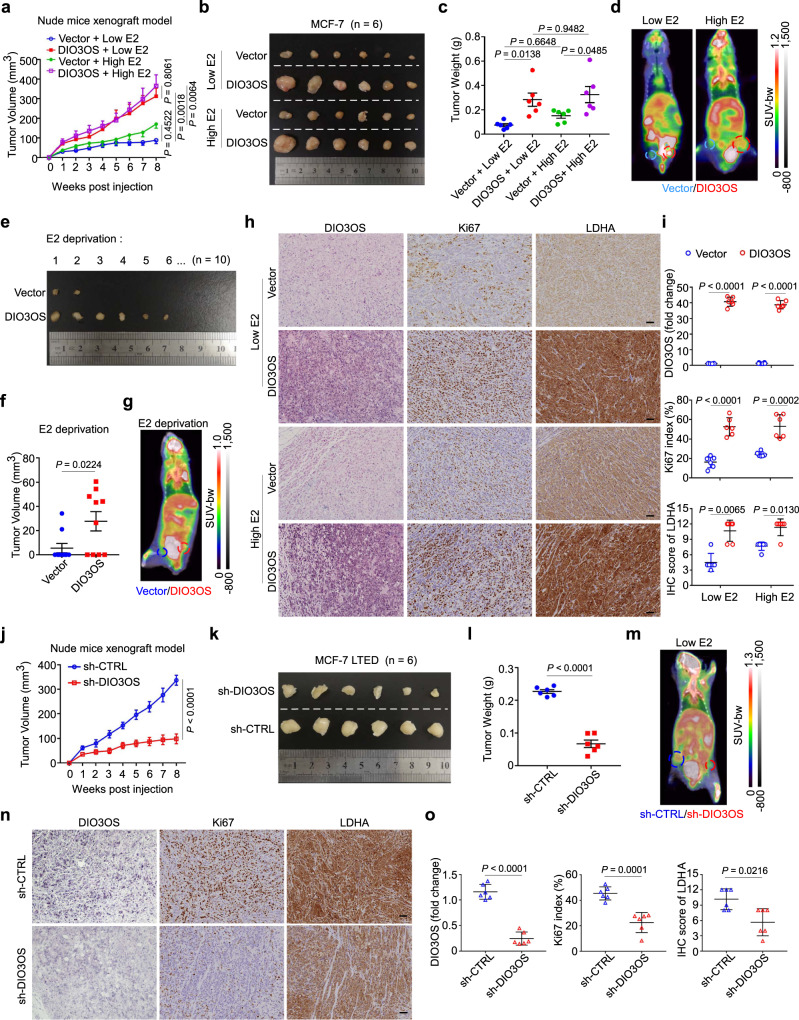
DIO3OS promotes ER-positive breast tumor progression in vivo. **a**–**c** Growth curves of tumor xenografts (**a**), tumor size (**b**), and tumor weight (**c**) in nude mice bearing parental MCF-7 cells stably transfected with control or DIO3OS-overexpressing plasmid. The mice were pretreated with low or high dose of estrogen tablets. **d** Representative PET–CT scan images of the glucose accumulation in tumor xenografts arisen from parental MCF-7 cells stably transfected with control or DIO3OS-overexpressing plasmid under low- or high-dose estrogen treatment. **e**, **f** Tumor size (**e**) and tumor volume (**f**) in mice orthotopically injected with parental MCF-7 cells harboring control or DIO3OS-overexpressing plasmid without estrogen tablet. **g** Representative PET–CT scan images of the glucose accumulation in tumor xenografts arisen from parental MCF-7 cells stably transfected with control or DIO3OS-overexpressing plasmid without estrogen treatment. **h**, **i** Representative ISH staining of DIO3OS and IHC staining for LDHA/Ki67 expression in indicated tumor xenografts arisen from parental MCF-7 cells (**h**) and the quantitative analyses (**i**). Scale bars, 50 μm. **j**–**l** Growth curves of tumor xenografts (**j**), tumor size (**k**), and tumor weight (**l**) in mice that were orthotopically injected with MCF-7 LTED cells transducing lenti-shDIO3OS or lenti-control. The mice were pretreated with low dose of estrogen tablets. **m** Representative PET–CT scan images of the glucose accumulation in tumor xenografts arisen from MCF-7 LTED cells transducing lenti-shDIO3OS or lenti-control. **n**, **o** Representative ISH staining of DIO3OS and IHC staining for LDHA/Ki67 expression in indicated tumor xenografts arisen from MCF-7 LTED cells (**n**) and the quantitative analyses (**o**). Scale bars, 50 μm. For **a**–**c**, **h**–**o**, *n* = 6 mice per group. For **e**, **f**, *n* = 10 mice per group. Each dot represents one mouse (**c**, **f**, **i**, **l**, **o**). Means ± s.e.m. (**a**, **c**, **f**, **j**, **l**) are presented, and *P*-values were analyzed using two-sided one-way ANOVA with Sidak’s multiple-comparisons test (**a**, **c**), two-tailed Student’s *t*-test (**f**, **j**, **l**). For **i**, **o**, means ± s.d. are shown, and *P*-values were determined using two-tailed Student’s *t*-test (for DIO3OS expression and Ki67 index), Mann–Whitney test (for IHC score of LDHA). For **d**, **g**, **m**, colored circles denoted the xenografts with indicated treatments. E2 estrogen. Source data are provided as a Source Data file.

To further explore whether DIO3OS regulates ER-positive breast cancer development in vivo, we orthotopically injected parental MCF-7 cells with or without DIO3OS overexpression in ovariectomized nude mice. These mice were previously divided into three groups by treating with different doses of estrogen tablets. In the low-dose (0.18 mg) estrogen-treated group, the tumor growth of DIO3OS-overexpressing MCF-7 xenografts was significantly promoted, compared with controls (Fig. 7a–c). Similarly, enforced DIO3OS expression accelerated the growth of tumor when conditioned with high-dose (0.72 mg) estrogen (Fig. 7a–c). Furthermore, under the condition of complete estrogen deprivation by ovariectomy and without estrogen tablet treatment, DIO3OS overexpression strikingly boosted the tumorigenicity in nude mice (6/10), in comparison to the control group (2/10) (Fig. 7e, f). Moreover, enhanced ^18^F-fluorodeoxyglucose (^18^FDG) accumulation was observed in tumor xenografts from mice with enforced expression of DIO3OS, regardless of estrogen administration, as assessed by positron emission tomography and computed tomography (PET–CT) scanning (Fig. 7d, g). In addition, xenografts with DIO3OS upregulation exhibited more intensive Ki67 staining than those with control DIO3OS expression, supporting the pro-tumor role of DIO3OS in vivo (Fig. 7h, i). Meanwhile, higher LDHA expression was observed in xenografts with DIO3OS overexpression, as detected by immunohistochemistry (Fig. 7h, i), which was consistent with the enhanced glycolytic level upon DIO3OS overexpression. These results showed that DIO3OS might promote the estrogen-independent growth of ER-positive breast tumors in vivo.

Conversely, DIO3OS knockdown suppressed the growth of MCF-7 LTED xenografts (Fig. 7j–l), reduced ^18^FDG accumulation (Fig. 7m) and decreased the expression of LDHA along with Ki67 in xenografts of ovariectomized nude mice with low-dose estrogen treatment (Fig. 7n, o). Together, DIO3OS positively regulates the estrogen-independent breast cancer cell proliferation and glucose metabolism in vivo.

## Discussion

Aerobic glycolysis is one of the hallmarks of metabolic reprogramming in human cancers. Here, we demonstrate that lncRNA DIO3OS directly interacts with PTBP1 protein in the nucleus and further stabilizes the mRNA of LDHA by protecting the integrity of its 3’UTR, ultimately activates glycolytic metabolism in AI-resistant breast cancer cells. LDHA is a pivotal glycolytic enzyme that converts pyruvate to lactate. As a crucial metabolite in glycolysis pathway, lactate not only fuels the energy supply for cancer growth, but also serves as a signaling molecule in promoting cancer angiogenesis, invasion and migration, as well as immune escape^3–6^. It has been documented that LDHA is upregulated in various human cancers, which promotes glycolytic metabolism and cancer development. Inhibition of LDHA activity can re-sensitize breast tumor cells to anti-HER2 therapy (trastuzumab) or chemotherapy (taxol)^36,37^.

Recently, lncRNAs have been reported to participate in LDHA regulation. HULC and LINC00973 can directly bind to LDHA protein and enhance its enzymatic activity in cancers^38,39^. LncRNA GLCC1 stabilizes c-Myc through direct interaction with HSP90 chaperon and transactivates target genes such as *LDHA*, thereby reprograming glycolytic metabolism for cancer cell proliferation^40^. Different from these studies, we discover that DIO3OS interacts with PTBP1 to modulate the stability of LDHA mRNA. Specifically, DIO3OS and PTBP1 can bind to and presumably protect the 3’UTR of LDHA mRNA from splicing-induced deletion, and thus maintain its integrity and stability. Most eukaryotic mRNA has a polyadenylic acid (poly A) tail downstream of 3’UTR, which is involved in the regulation of mRNA stability and transport^41,42^. Selective usage of alternative poly(A) sites residing in 3’UTR has been reported to mediate 3’UTR shortening, without altering the coding capacity of the mRNA^43^. As the well documented types of alternative poly(A) signals are located within the 3’UTR of LDHA mRNA, alternative polyadenylation (APA) seems not adequate to account for the complete absence of 3’UTR in LDHA-220. In fact, AS within UTRs has been found in the transcript variants of at least 13% of genes^44,45^. Alternative 3’UTR formation can also be mediated by a dual mechanism, which involves both APA and AS^44,46^. Reciprocally, lacking 3’UTR may hinder the formation of poly A tail and cause aberrant mRNA processing^45,47^. In our study, knockdown of DIO3OS or PTBP1 contributes to a splicing switch from canonical LDHA transcript to an LDHA variant lacking 3’UTR, which is proven to be unstable. Overexpression of canonical transcript LDHA-203, but not the unstable transcript LDHA-220, markedly enhances lactate production and promotes breast cancer cell growth. Thus, our study uncovers a mechanism of regulating LDHA at the mRNA level which depends on splicing switch induced by lncRNA DIO3OS expression.

The third-generation AI therapy has become the standard of care for ER-positive postmenopausal breast cancer patients, yet resistance to AI treatment remains an important clinical challenge^12–14^. Previous studies have revealed mechanisms of intrinsic and acquired resistance to AI therapy, most of which involves the deregulation of the ER pathway. It has been reported that MCF-7 LTED cells display higher aerobic glycolytic activity than their parental counterparts, which is mechanistically regulated by ER-dependent miR-155 expression^48^. Although we also detected enhanced aerobic glycolysis in breast cancer cells that have undergone LTED, the mechanism was not linked to ER. Instead, we have characterized a lncRNA DIO3OS that remodels glycolytic metabolism in AI-resistant cancer cells. As we and others have found that AI-resistant breast cancer may rely more on aerobic glycolysis for tumor growth, DIO3OS confers growth advantages to AI-resistant cells by regulating splicing switch to enhance aerobic glycolysis. These results highlight a critical role of DIO3OS in inducing AI resistance by activating an alternative proliferating pathway independent of ER.

Although estrogen deprivation can mimic the hormone withdrawal that occurs during AI treatment in clinic, this in vitro model cannot simulate the adaptive changes within cancer cells chronically treated with an AI, nor can it be employed to detect the regulatory effects of AI drugs on aromatase expression and activity. This is because there is low or no endogenous aromatase expressed in breast cancer cell lines. To solve this problem, other research groups have used breast cancer cells stably overexpressing the human aromatase gene to study AI response. However, this model cannot account for the fact that it is the stromal cells of breast cancers that predominantly express aromatase to convert androgens to estrogens^49,50^. As such, we have detected the expression level of DIO3OS in tumor samples from ER-positive breast cancer patients receiving AI therapy, and have demonstrated its prognostic value in these patients, further confirming the clinical relevance of above-mentioned findings.

Nuclear lncRNAs often exert their biological functions by regulating gene expression in cis or in trans. We demonstrate that lncRNA DIO3OS functions through the interaction with PTBP1 protein in the nucleus. PTBP1 is a splicing suppressor, which can compete with spliceosome for RNA binding and thereby inhibits alternative splicing^28,30,32^. As lncRNAs can act as molecular scaffolds to bring together larger complexes and localize splicing regulators, the DIO3OS-PTBP1 interaction may be important for the nuclear localization and function of PTBP1. Various studies have reported that PTBP1 regulates oncogenic splicing switch in pyruvate kinase from splice variant PKM1 towards PKM2, thereby contributing to energy metabolism remodeling from oxidative phosphorylation to aerobic glycolysis, as well as resistance to cancer therapy^51,52^. A neural-specific lncRNA named Pnky can also interact with PTBP1 to regulate the alternative splicing of a core set of transcripts which are involved in the neuronal differentiation and neurogenesis^31^. To explore the alteration of splicing events induced by DIO3OS and PTBP1 in LTED cells, we identified the concordantly occurred AS events in DIO3OS-knockdown and PTBP1-knockdown cells, which occurred in genes pointed to glycolysis pathway. Screen of the glycolytic metabolites affected by DIO3OS and PTBP1 and further verification of the splicing events in glycolytic enzymes identified *LDHA* as a key downstream target of DIO3OS and PTBP1 in AI-resistant breast cancer cells.

Recently, genetic polymorphisms (rs10420407) in the *PTBP1* gene were found to affect the patient response to androgen-deprivation therapy in prostate cancer^53^. So far, no breast cancer-associated single nucleotide polymorphisms (SNPs) of PTBP1 have been reported, however, this finding may have implications for the role of PTBP1 SNP in endocrine therapies in cancers.

Targeting cancer metabolism emerges as an important strategy to improve anti-cancer therapy. As the expression of DIO3OS increases in breast cancer cells undergoing AI resistance, the aberrant activation of DIO3OS/PTBP1/LDHA glycolysis cascade may endow tumor cells with metabolic adaptation that allows them to survive from AI-caused estrogen deficiency. Future translational studies will focus on exploring effective approach for specific DIO3OS knockdown to re-sensitize breast cancer cells to AI treatment.

## Methods

### Study approval

All tumor samples from breast cancer patient used in this study were obtained under informed written consent and approval of the Internal Review and Ethics Board of Sun Yat-Sen Memorial Hospital, Sun Yat-Sen University, according to ethical regulations. In vivo experiments in murine model were reviewed and approved by the Institutional Animal Care and Use Committee (IACUC) guidelines at Sun Yat-Sen University (SYSU-IACUC-2020-000062).

### Cell culture and treatment

BT549, MDA-MB-231, MDA-MB-468, Hs578T, MDA-MB-436, SKBR3, BT474, MDA-MB-361, MCF-7, and T47D human breast cancer cell lines were obtained from the American Type Culture Collection (ATCC) and cultured according to standard protocols. All the cell lines were authenticated by short tandem repeat profiling prior to use and tested negative for mycoplasma contamination. For the establishment of the AI-resistant cell lines, MCF-7 and T47D cells were grown in steroid-deprived phenol red-free RPMI 1640 medium (Gibco™) with 10% dextran charcoal-stripped (DCC) fetal bovine serum (Hyclone) for at least 10 months to gain AI resistance, termed as LTED cells. For functional experiments, cells were cultured for 3–7 days in DCC medium. For the assessment of AI drug sensitivity, LTED cells were changed with fresh medium supplemented with 10^−8^–10^−5^ mol/L letrozole (Sigma) and anastrozole (Sigma) every 2 days and counted at 8^th^ day using an automated cell counter (Countstar IC1000). Fulvestrant (Selleck) at a final concentration of 1 μM in the ordinary culture medium was used to induce ER downregulation in LTED cells.

### Seahorse assay

In vitro cellular metabolic alterations were accessed by Seahorse XF24 extracellular flux analyzer (Seahorse Bioscience), following the manufacturer’s instructions. Briefly, MCF-7/T47D cells with indicated treatments were seeded in XF24-well culture plates at a density of 3000 (MCF-7) or 5000 (T47D) cells per well overnight, followed by a 24 h serum starvation. After 1 h incubation with basal medium containing 2 mM L-glutamine at 37 °C, cells were subjected to assessment of the extracellular acidification rates (ECAR) or oxygen consumption rate (OCR) at 8 min intervals. For the measurement of ECAR, glucose, oligomycin, and 2-deoxyglucose (2DG) was sequentially injected to the wells at every interval time of 3 measurement, with a final concentration of 10 mM, 1 μM, and 50 mM, respectively. In terms of the OCR detection, oligomycin, carbonyl cyanide-4 (trifluoromethoxy) phenylhydrazone (FCCP), and rotenone/antimycin A were in turn added to the wells after every 3 measurements at intervals of 8 min, with a final concentration of 1 μM, 0.5 μM, or 0.5 μM, respectively. The reading difference between the ECAR following oligomycin addition and the basal ECAR was represented as the glycolytic capacity.

### Glucose uptake, lactate production, and acetyl-CoA level measurement

Cells with indicated treatments in 96-well plates were grown to about 40% confluence and replenished with fresh medium. Twenty-four hours later, Glucose Uptake Assay Kit (#ab136955, Abcam), Lactate Colorimetric Assay Kit II (#K627-100, BioVision) or PicoProbe™ Acetyl CoA Assay Kit (#K317-100, BioVision) was employed to measure the glucose uptake, lactate production or acetyl-CoA level following the manufacturer’s instructions, respectively.

### Liquid chromatography-mass spectrometry (LC-MS) analysis

For metabolites analysis by LC-MS, cells with indicated treatments were seeded in six-well plates at ~80% confluence. With the medium aspirated and the plates rinsed thrice with ice-cold saline, the plates were placed on liquid nitrogen to quench cell metabolism, followed by addition of 1 mL of pre-cooled (−80 °C) 80% (vol/vol) methanol containing internal standards and incubation at −80 °C for 20 min. The cell lysate/methanol mixture was scraped and collected into a 1.5 mL conical tube, followed by addition of 500 μL of pre-cooled (−80 °C) acetonitrile/water (80:20, vol/vol) on the culture plates, which was transferred to the same conical tube. After a 10-min centrifugation (18,000 × *g* at 4 °C), the supernatant was transferred to another 1.5 mL conical tube and dried with SpeedVac. The analysis of all and samples were performed on ultra-performance liquid chromatography coupled to electrospray ionization tandem mass spectrometry platform (UPLC–ESI-MS/MS; Agilent 1290 Infinity II with Agilent G6495A). Mobile phase A and mobile phase B were LC/MS-grade water and 85% acetonitrile respectively that both contained 20 mM ammonium acetate and 0.1% ammonium hydroxide (35% (wt/vol). Dried samples were resuspended in mobile phase (A/B, 4/6) and 3 μL was injected and chromatographically separated on an amide XBridge HPLC column (4.6 mm inner diameter × 100 mm length; 3.5 μm; Waters) maintained at 40 °C, the target metabolites were eluted with a 92–50% gradient of mobile phase B at flow rate of 0.6 mL/min. Mass spectral data were acquired in a positive/negative ion switching mode with multiple reaction monitoring of precursor and characteristic product ions specific to each metabolite. These metabolites were identified according to standard compounds and integrated using the Agilent MassHunter workstation QQQ quantitative analysis software (Version B.07.00). Results of each sample were normalized based on total protein concentration.

### Patient tissue specimens

Tumor samples (*n* = 257) were obtained from female patients diagnosed with ER-positive breast cancer, receiving AI treatment post surgical resection at Sun Yat-Sen memorial Hospital, Sun Yat-Sen University ranging from 2011 to 2018 with informed written consents. All study-related procedures were conducted under the approval and guidance from the Internal Review and Ethics Board of Sun Yat-Sen Memorial Hospital. Acquired endocrine resistance of breast cancer refers to those recurrence and metastasis after receiving more than two years of postoperative endocrine therapy, or recurrence and metastasis within one year after finishing adjuvant endocrine therapy, or disease progression of metastatic breast cancer after receiving the first-line endocrine therapy more than six months.

### RNA extraction and quantitative real-time PCR (qRT-PCR)

RNA was extracted by TRIzol agent (Invitrogen), homogenized in chloroform, and then purified by isopropanol and ethanol. Frozen tissues in TRIzol were subjected to mechanical homogenization by a TissueLyserII (Qiagen), with a 5-mm stainless steel bead per sample. For qPCR, RNA concentration and quality were assessed by the NanoDrop 2000 Spectrophotometer (Thermo Fisher Scientific). At most 1 μg of total RNA per sample was reverse transcribed with a PrimeScript™ RT Reagent Kit (#RR014A, Takara), subsequent real-time qPCR amplification was performed on a LightCycler 480 instrument (Roche) with SYBR Premix Ex Taq^TM^ (#RR420L, TAKARA). Gene expression level was normalized by GAPDH or ACTB. All primer sequences in this study are included in Supplementary Table 5.

### High-throughput RNA sequencing

Total RNA was extracted using TRIzol (Invitrogen) according to the manufacturer’s instructions. Both integrity and concentration of RNA was assessed with the RNA Nano 6000 Assay Kit of the Bioanalyzer 2100 system (Agilent Technologies, USA). A total of 2 μg RNA per sample was used and the sequencing libraries were generated with NEBNext® Ultra™ RNA Library Prep Kit for Illumina® (NEB, USA). Sequencing was performed on an Illumina platform (Annoroad Gene Technology Co., Ltd., China) and 150 bp paired-end reads were generated, mapped to the human reference genome (hg38 sourced from UCSC Genome Browser) with HISAT2 v2.1.0. Reads counts for genes in each sample were assessed by HTSeq v0.6.0, and fragments per kilobase per million mapped reads (FPKM) was calculated to estimate gene expression in each sample. Genes with *P-*value ≤ 0.05 and |log2_ratio| ≥ 1 are identified as differentially expressed genes (DEGs). Heatmap and volcano plot were generated by using R package. The sequencing data have been deposited in the National Center for Biotechnology Information (NCBI) Gene Expression Omnibus (GEO) public database under accession code GSE206199 and GSE206142. For Gene Set Enrichment Analysis (GSEA), software of GSEA v.4.2.3 was accessible from the GSEA website (http://www.broadinstitute.org/gsea). For KEGG analysis, the DAVID online bioinformatics system (https://david.ncifcrf.gov) was utilized.

### Northern blot

Total RNA premixed with RNA loading buffer was loaded onto 2% formaldehyde–MOPS–agarose gels, separated by electrophoresis and then transferred to a nylon membrane (GE Healthcare) for over 6 h. Subsequently, the blotted membrane was prehybridized at 52 °C with the DIG Easy Hyb buffer (#11796895001, Roche) for 30 min, followed by further incubation with a digoxigenin (DIG)-labeled DIO3OS LNA oligonucleotides (5DiGN/CCCAGAACAGATGACAGCCCT/3DiG_N; ACTB, 5DiGN/CTCATTGTAGAAGGTGTGGTGCCA/3DiG_N; Qiagen) overnight. The probe–target hybrids were detected by the DIG Luminescent Detection kit (#11363514910, Roche).

### Rapid amplification of cDNA ends (RACE)

The 5’/3’ RACE of DIO3OS RNA was determined using the SMARTer RACE 5’/3’ Kit (#634860, Clontech) following the manufacturer’s guidance. The RNA template for DIO3OS was extracted from MCF-7 LTED cells and all gene-specific primers used in RACE are listed in Supplementary Table 5.

### RNA decay assay

Cells with indicated treatments were plated into six-well plates. The mRNA decay rates were estimated by inhibiting the mRNA transcription with 10 µg/mL actinomycin D (APExBio) in culture medium. The equal volume of DMSO was used for negative control. RNA was extracted at indicated time points for further qRT-PCR analysis, as the relative amount of specific mRNA remaining in each sample was linked to mRNA degradation. Results were normalized to the expression level of reference gene GAPDH in each sample.

### In vitro translation assay

For the generation of putative DIO3OS-GFP fusion protein, the full-length sequence of DIO3OS was amplified by reverse transcription PCR and then cloned into the mammalian expression vector pcDNA3.1 with a GFP fused to the carboxyl-terminus. After validation by sanger sequencing, the fusion plasmids were used for cell transfection.

### Transfection and transduction of tumor cells

The LNAs antisense oligonucleotides (Exiqon) and siRNA/shRNA (Genepharma) sequences are listed in Supplementary Table 6. DIO3OS and GFP-DIO3OS fusion proteins, FLAG-tagged full-length and truncated PTBP1 constructs, LDHA variants, luciferase-LDHA 5’/3’UTR chimeric PCDNA3.1 plasmid as well as pGL3 luciferase reporter plasmid used for ectopic expression in cell lines were constructed by Generay Biotech. Cells were transfected with indicated LNAs, siRNAs, or plasmids using Lipofectamine 3000 (#L3000008, Invitrogen) following the manufacturer’s recommendations. For the screening of stable DIO3OS-overexpressed cells, cells were changed fresh medium supplemented with 1 mg/ml G418 every 2 days for at least two weeks. For transduction of tumor cells, cells were seeded into six-well plates to ~40% confluence and transduced with lentiviral particles at an estimated multiplicity of infection of 5 with 8 µg/mL polybrene (Sigma). To select stable DIO3OS-knockdown cells, 5 µg/mL puromycin (Gibco) was used 72 h after transfection.

### EdU incorporation assay

The EdU incorporation assay was conducted using a Cell-Light EdU Apollo®567 In Vitro Imaging Kit (#C10310-1; Guangzhou RiboBio Co., Ltd.). Briefly, cells with indicated treatment were incubated with diluted EdU reagent (1:5000 in complete culture medium) at 37 °C overnight. After fixation with 4% paraformaldehyde (PFA), cells were subjected to 30 min incubation with Apollo Staining reaction liquid at room temperature, protected from light. Hoechst 33342 reagent was applied to counterstain the nuclei for another 30 min and the images were obtained from an automatic inverted fluorescence microscope (Axio Observer Z1, Zeiss).

### RNA pulldown assay

Biotin-16-UTP labeled DIO3OS RNA was in vitro transcribed with a MEGAscript™ T7 Transcription Kit (#AM1333, Thermo Fisher Scientific) following the manufacturer’s guidance. Briefly, 3 ug of folded biotin-RNA was incubated with 1 mg of total protein extract in IP lysis buffer (#87788, Thermo Fisher Scientific) under 1 h gentle rotation. Afterward, the mixture was incubated with 50 µL of prewashed Dynabeads® M-280 Streptavidin (#11205D, Thermo Fisher Scientific) at room temperature for another hour. After extensive washing with IP lysis buffer using a magnetic stand, the retrieved protein was detected by either polyacrylamide gel-electrophoresis (PAGE) or immunoblot. The specific silver-stained protein bands cut out of PAGE gel were subjected to further identification by liquid chromatographic-tandem mass spectrometric.

### RNA immunoprecipitation and crosslinking immunoprecipitation (CLIP)

RNA immunoprecipitation assay was conducted with Magna RIP™ RNA-Binding Protein Immunoprecipitation Kit (#17-700, Millipore), following the manufacture’s protocol. In brief, cells were lysed by RIP lysis buffer supplemented with RNase inhibitor (#N2111S, Promega) and protease inhibitor (#8444, Thermo Fisher Scientific), and incubated at 4 °C with antibody-conjugated Protein A/G Dynabeads overnight. Specific anti-PTBP1 antibody (#57246, CST, dilution 1:100) and negative control rabbit IgG (#17-700, Millipore, 5 μg per reaction) were used for the retrieval of RNA. After rigorous washes with RIP lysis buffer, co-precipitated RNA was detected by qRT-PCR analysis. For CLIP assay, before cell lysis, 0.3% formaldehyde (in PBS) was added to the cell culture plates for 10 min, shaking at room temperature, followed by incubation with 0.125 M glycine for 5 min to stop crosslinking.

### Immunoblot

Total protein was extracted from cultured cells using RIPA lysis buffer supplemented with a protease and phosphatase inhibitor cocktail (#78444, Thermo Fisher Scientific), quantified with the Pierce BCA Protein Assay kit (#23225, Thermo Fisher Scientific), electrophoresed through SDS–polyacrylamide gels, and transferred onto polyvinyldifluoride (PVDF) membranes (#03010040001, Roche). Primary antibodies (dilution 1:1000) against ER (#8644, CST), phospho-ER(Ser118) (#2511, CST), phospho-ER(Ser167) (#64508, CST), PTBP1 (#57246, CST), LDHA (#3582, CST), HSP90 (#4877, CST), FLAG (#F1804, Sigma), and horseradish peroxidase (HRP)-conjugated antibodies (dilution 1:10000) against β-actin (#HRP-66009, Proteintech), GAPDH (#HRP-60004, Proteintech), α-tubulin (#HRP-66031, Proteintech), as well as peroxidase-conjugated secondary anti-rabbit or mouse antibody (#7074 or #7076, CST) were utilized. The antigen–antibody reaction was visualized using enhanced chemiluminescence assay (#34577, Thermo Fisher Scientific). Immunoblot images have been cropped for presentation, with positions of the molecular mass marker (kDa) indicated alongside each blot. The intensity of individual bands on immunoblots was measured by ImageJ software (v.1.43), and plotted as normalized values relative to the loading control. Uncropped scans of all blots are provided in the Source Data file and the Supplementary Information.

### Luciferase reporter assay

pGL3 luciferase reporter plasmid carrying estrogen receptor element (ERE) sequence or pcDNA3.1 plasmids fused with luciferase-LDHA-5’/3’UTR chimeric RNA were co-transfected with the pRL-TK Renilla control construct (Promega) into cultured cells. Firefly luciferase activities were assayed by the Dual-Luciferase Reporter Assay System (#E1910, Promega), and normalized to the Renilla luciferase activity.

### Fluorescence in situ hybridization (FISH) and in situ hybridization (ISH)

For FISH of DIO3OS and colocalization analysis of DIO3OS and PTBP1, cells were fixed with 4% PFA for 20 min at room temperature and then permeabilized using 0.5% Triton X-100 in PBS for 10 min on ice. After 2 h prehybridization, the cell-adherent slides were hybridized by DIG-labeled LNA DIO3OS probe (final concentration at 25 nm in the hybridization solution; Qiagen) at 52 °C overnight, followed by incubation with anti-DIG-fluorescein (green, #11207741910, Roche, dilution 1:100) and anti-PTBP1 (#57246, CST, dilution 1:100) antibodies at 4 °C overnight. Alexa Fluor 555–conjugated secondary antibody (red, #A-21428, Thermo Fisher Scientific, dilution 1:200) was used for signal visualization. With nuclei counterstained by Hoechst 33342 (#H21492, Thermo Fisher Scientific), high-resolution fluorescent images were acquired on the confocal laser scanning microscopes at 63 × magnification (LSM780/800, Zeiss) and processed with ZEN 2012 Lite software.

For RNA ISH, sections of the paraffin-embedded tissue samples were dewaxed and rehydrated following standard protocols. After 30 min digestion with 5% trypsin, the tumor sections were subjected to fixation in 4% PFA at room temperature and 2 h prehybridization with hybridization solution at 52 °C. Afterward, a 25 nM DIG-labeled probe targeting DIO3OS was used for hybridization at 52 °C overnight in a humidified chamber. With the nuclei counterstained with Nuclear Fast Red solution, ISH signals were detected as blue-purple-colored staining under an Olympus BX51 microscope (Olympus, Tokyo, Japan). The staining index (SI) of DIO3OS was calculated as the multiplication of the proportion and intensity scores of positively stained cells by counting at least 10 random fields (objective ×20)^54^. In detail, the proportion of DIO3OS positively stained tumor cells was graded into five levels (0, no positive cells; (1) <25% DIO3OS-positive cells; (2) 25–50% DIO3OS-positive cells; (3) 50–75% DIO3OS-positive cells; (4) >75% DIO3OS-positive cells). The staining intensity was scored on a four-point scale (0, no DIO3OS staining; 1, weak DIO3OS staining; 2, moderate DIO3OS staining; 3, strong DIO3OS staining). The expression of DIO3OS was recorded from 0 to 12 according to the SI, with an optimal cutoff value of <3 (low) versus ≥ 3 (high).

### Immunohistochemistry staining

Sections of the paraffin-embedded tumor samples were deparaffinized, rehydrated, followed by antigen retrieval. Endogenous peroxidase was eliminated by 3% hydrogen peroxide. The tissue sections were blocked with goat serum for 30 min at room temperature and then incubated with primary antibody against LDHA (#3582, CST, dilution 1:200) or anti-Ki67 (#ZM-0166, ZSGB-BIO, ready to use) reagent overnight at 4 °C. The immunodetection was performed with DAB (GK500710, Gene Tech) following the manufacturer’s instructions. The immunostaining was observed under an Olympus BX51 microscope (Olympus, Tokyo, Japan). The quantification of Ki67-positive cells was assessed using ImageJ software (v.1.43) with at least 5 ×200 magnification images per section^55^. The staining scores of LDHA-positive cells were calculated according to their proportion and intensity in 10 random fields (objective ×20)^6^. The evaluation of automated measurements was reviewed independently by two pathologists.

### Animal experiments

Four**-**week-old female athymic BALB/c nude mice purchased from the Vital River Laboratories (Beijing) were raised under standard conditions (20–26 °C temperature, 40–60% humidity) with a 12 h light/12 h dark cycle at the specific-pathogen-free (SPF) animal facility in the Laboratory Animal Resource Center of Sun Yat-Sen University until six weeks old. Mice were initially ovariectomized and allowed to acclimatize for a period of seven days before subcutaneous estrogen tablet implantation. Six to ten mice were randomly assigned to each group for different treatments. Another week later, a total of 0.1 mL sterile cell suspension containing 1 ×10^7^ cells with or without altered DIO3OS expression in Matrigel (BD Biosciences) were injected orthotopically into the fourth pair mammary fat pads of nude mice. The monitoring of tumor volumes (mm^3^) was performed every week according to the formula width^2^ × length / 2. Maximum tumor size (1500 mm^3^) permitted by ethics committee was not exceeded. Humane endpoints in this study were also referred to cachexia, body-weight reduction of 10% or infection, etc. At the experimental endpoint, mice were euthanized by cervical dislocation following overdose anesthesia.

### Positron emission tomography/computed tomography imaging

Glucose accumulation of nude mice bearing breast tumor xenografts at 8^th^ week was detected using the Inveon micro-PET/CT Scanner (Siemens). Prior to micro-PET scanning, all mice were fasted for at least 8 h, followed by anaesthetization, and then received 5 µCig^−1^ [^18^F] fluorodeoxyglucose (^18^F-FDG) in saline (100 μL) intravenously via tail vein injection. Forty minutes later, a static 15 min PET scan was performed for the static acquisition. Collected images were further corrected for attenuation, scatter, normalization and camera dead time, followed by co-registration with micro-CT images using the Inveon Research Workplace software under the manufacturer’s instructions. Tumor uptake of ^18^F-FDG measured in the three-dimensional regions of interest (ROIs) was determined according to the standardized uptake value (SUV).

### Statistics

All statistical analyses in this study were conducted by Microsoft Excel 2016, GraphPad Prism 8.0, SPSS version 20.0, R (v.4.0.3) and RStudio (v.1.3.1093). Exact n numbers, number of independent experiments with similar results, statistical tests and *P*-values were stated in figures and figure legends. Error bars in figures denote s.d. or s.e.m. Generally, all experiments were performed at least three times independently. Statistical differences between two groups were analyzed with two-tailed Student’s *t*-tests or Mann–Whitney test (for the quantification of ISH and IHC staining), while comparisons among more than two groups were performed using two-sided one-way ANOVA with Dunnett’s or Sidak’s multiple-comparisons test. All clinical and pathologic data were retrospectively collected from medical records and analyzed. The ISH/IHC staining of tumor tissue samples was quantified by research staff blinded to the clinical data and experimental design. Cutoff value for clinical sample grouping based on the DIO3OS staining scores in AI-treated breast cancer tissues was determined by X-tile software (Version 3.6.1, Yale University). Survival data were plotted as Kaplan–Meier curves and analyzed with log-rank test. The chi-square (*χ*2) test was used for comparing difference of frequencies among groups. *P*-value <0.05 was considered statistically significant.

## Supplementary information

Supplementary Information

Description of Additional Supplementary Files

Supplementary Data 1

Supplementary Data 2

## Data Availability

The high-throughput sequencing data generated in this study have been deposited in the NCBI-GEO public database under accession code GSE206199 and GSE206142. The isoform-level expression analysis of LDHA, and related sequences were obtained from the ISOexpresso database (http://wiki.tgilab.org/ISOexpresso/) and the UniProtKB database (https://www.uniprot.org/uniprotkb/P00338/entry), respectively. A reporting summary for this article is available as a Supplementary Information file. The remaining data are available within the Article, Supplementary Information or Source Data file. Source data are provided with this paper.

## Source data

Source Data
