# Supplementary material for: Long noncoding RNA DIO3OS induces glycolytic-dominant metabolic reprogramming to promote aromatase inhibitor resistance in breast cancer
Source: Nat Commun. 2022 Nov 22;13:7160. doi: 10.1038/s41467-022-34702-x (PMC9684133; doi:10.1038/s41467-022-34702-x)
Supplement: Supplementary file 1 — Supplementary Information [file 41467_2022_34702_MOESM1_ESM.pdf]

Long noncoding RNA DIO3OS Induces Glycolytic-dominant Metabolic Reprogramming to Promote Aromatase Inhibitor Resistance in Breast Cancer

Xueman Chen[#], Rong Luo[#], Yunmei Zhang[#], Shuying Ye, Xin Zeng, Jiang Liu, Di Huang, Yujie Liu, Qiang Liu, Man-Li Luo[*], Erwei Song[*]

[#] These authors contributed equally to this work.

[*] Correspondence to Man-Li Luo (luomli@mail.sysu.edu.cn) and Erwei Song (songew@mail.sysu.edu.cn)

Supplementary information

Contents

Supplementary Figure 1-6

Supplementary Table 1-6

Uncropped Blots and Gels

Supplementary Note 1

Supplementary Fig. 1

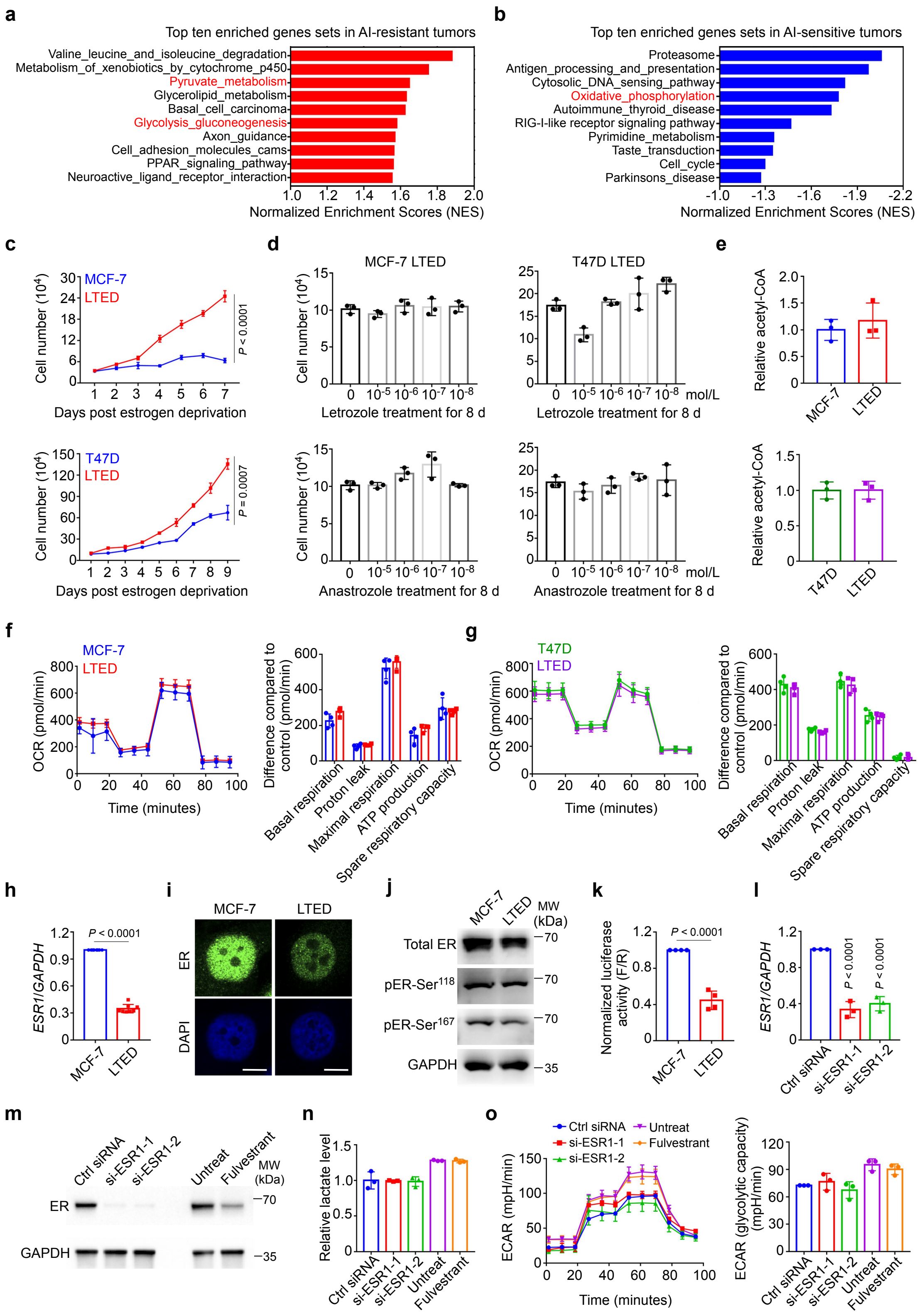

Supplementary Fig. 1 | AI-resistant ER-positive breast cancer exhibits enhanced aerobic glycolysis.

a,**b**, The top ten positively enriched (**a**) and top ten negatively enriched (**b**) gene sets in AI-resistant samples (relative to the AI-sensitive group) are displayed with their corresponding normalized enrichment scores (NES). **c**, Growth curves of MCF-7/T47D LTED cells compared with parental MCF-7/T47D cells. **d**, Cell number counting of MCF-7/T47D LTED cells treated with letrozole or anastrozole for 8 days. **e**, The acetyl-CoA production of MCF-7/T47D LTED cells in comparison with parental MCF-7/T47D cells that had undergone one-week estrogen deprivation. **f**,**g**, OCR values of MCF-7/T47D LTED cells compared with their parental cells that had undergone one-week estrogen deprivation. **h**, qRT–PCR of the ESR1 mRNA level in parental and LTED MCF-7 cells. **i**, Representative immunofluorescence images of ER expression in MCF-7 LTED cells in comparison with MCF-7 cells. Scale bars, 10 μm. **j**, Immunoblot of the expression of total ER and phosphorylated ER in MCF-7 LTED cells in comparison with MCF-7 cells. **k**, Luciferase reporter assay of the basal transcriptional level of ER in MCF-7 LTED cells compared with MCF-7 cells. **l**, qRT–PCR of ESR1 knockdown efficiency in MCF-7 LTED cells by indicated siRNAs. **m**, Immunoblot of ER knockdown efficiency in MCF-7 LTED cells by indicated siRNAs and fulvestrant treatment. **n**, Lactate production of MCF-7 LTED cells with ER knockdown by indicated siRNAs or fulvestrant treatment. **o**, ECAR values and calculated glycolytic capacity of MCF-7 LTED with ER knockdown by indicated siRNAs or fulvestrant treatment. Means ± s.d. of n = 3 (**c**-**e**,**l**,**n**,**o**), n = 4 (**f**,**g**,**k**) or n = 8 (**h**) independent experiments yielding similar results, one representative experiment out of three that were similar (**i**,**j**,**m**) are shown, and *P* values were analyzed using two-tailed Student's *t*-test (**c**,**e**-**h**,**k**), two-sided one-way ANOVA with Dunnett's multiple-comparisons test (**d**,**l**). MW, molecular weight. Source data are provided as a Source Data file.

Supplementary Fig. 2

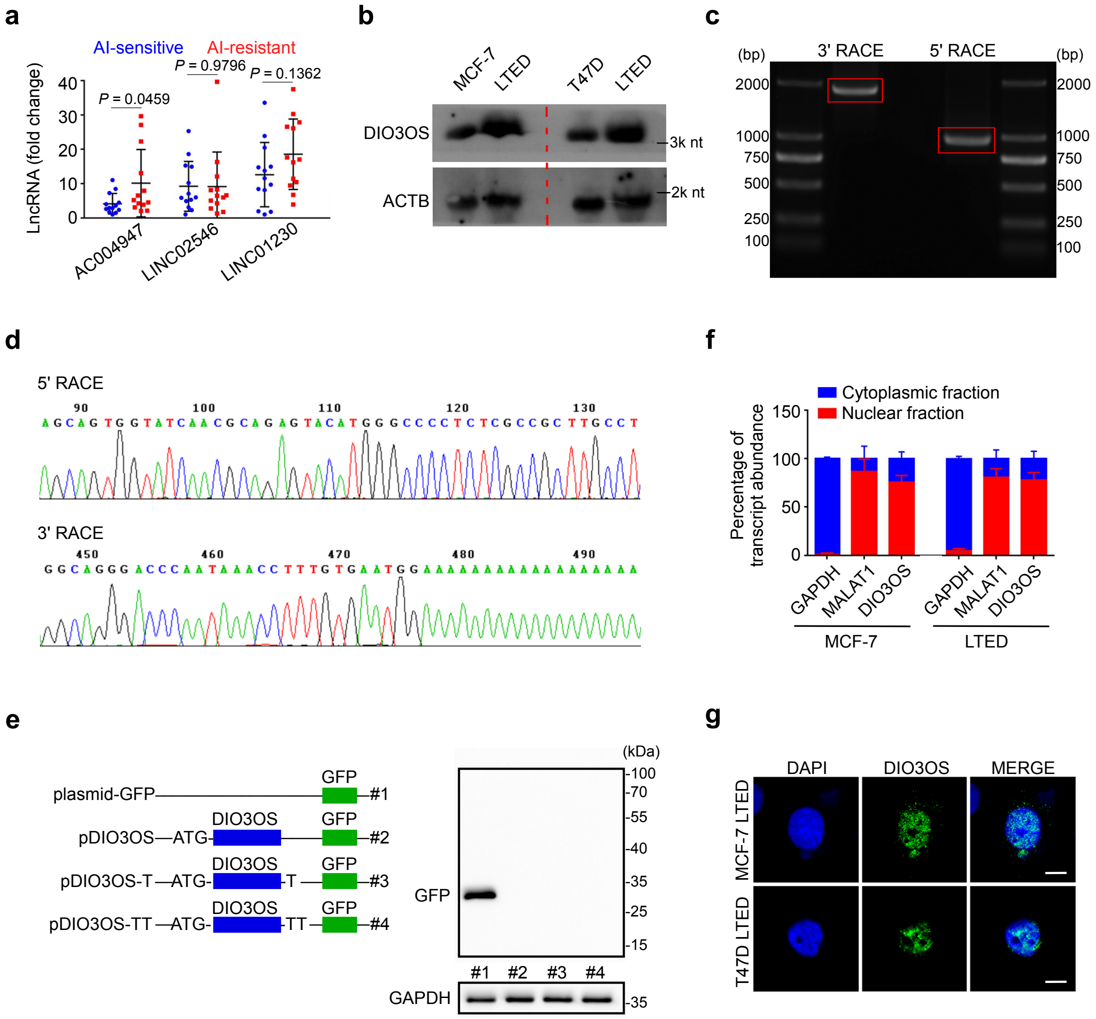

Supplementary Fig. 2 | AI-resistant ER-positive breast cancer exhibits upregulated DIO3OS expression.

a, qRT–PCR of candidate lncRNAs in tumor samples from ER-positive breast cancer patients that were sensitive or resistant to AI treatment. **b**, Northern blot of DIO3OS expression in MCF-7/T47D LTED cells compared with their parental cells. ACTB serves as loading control RNA. nt, nucleotide. **c**,**d**, 3'-/5'-RACE of DIO3OS (**c**) and sanger sequencing of the corresponding bands (**d**). **e**, Full-length DIO3OS was cloned into a eukaryotic expression vector pcDNA3.1 with an N-terminal codon ATG in three expression patterns. GFP serves as a positive control. **f**, qRT–PCR for DIO3OS expression in nuclear and cytoplasmic fractionations of parental and LTED MCF-7 cells. MALAT1 serves as a positive control for nuclear gene expression, while GAPDH RNA serves as the positive control for cytoplasmic gene expression. **g**, Representative FISH images of the nuclear localization of DIO3OS in MCF-7/T47D LTED cells. Scale bars, 10 μm. Means ± s.d. of n = 13 biological replicates (**a**) or n = 4 independent experiments (**f**), one representative experiment out of three that were similar (**b**,**c**,**e**,**g**), and all *P* values were analyzed using two-tailed Student's *t*-test. Source data are provided as a Source Data file.

Supplementary Fig. 3

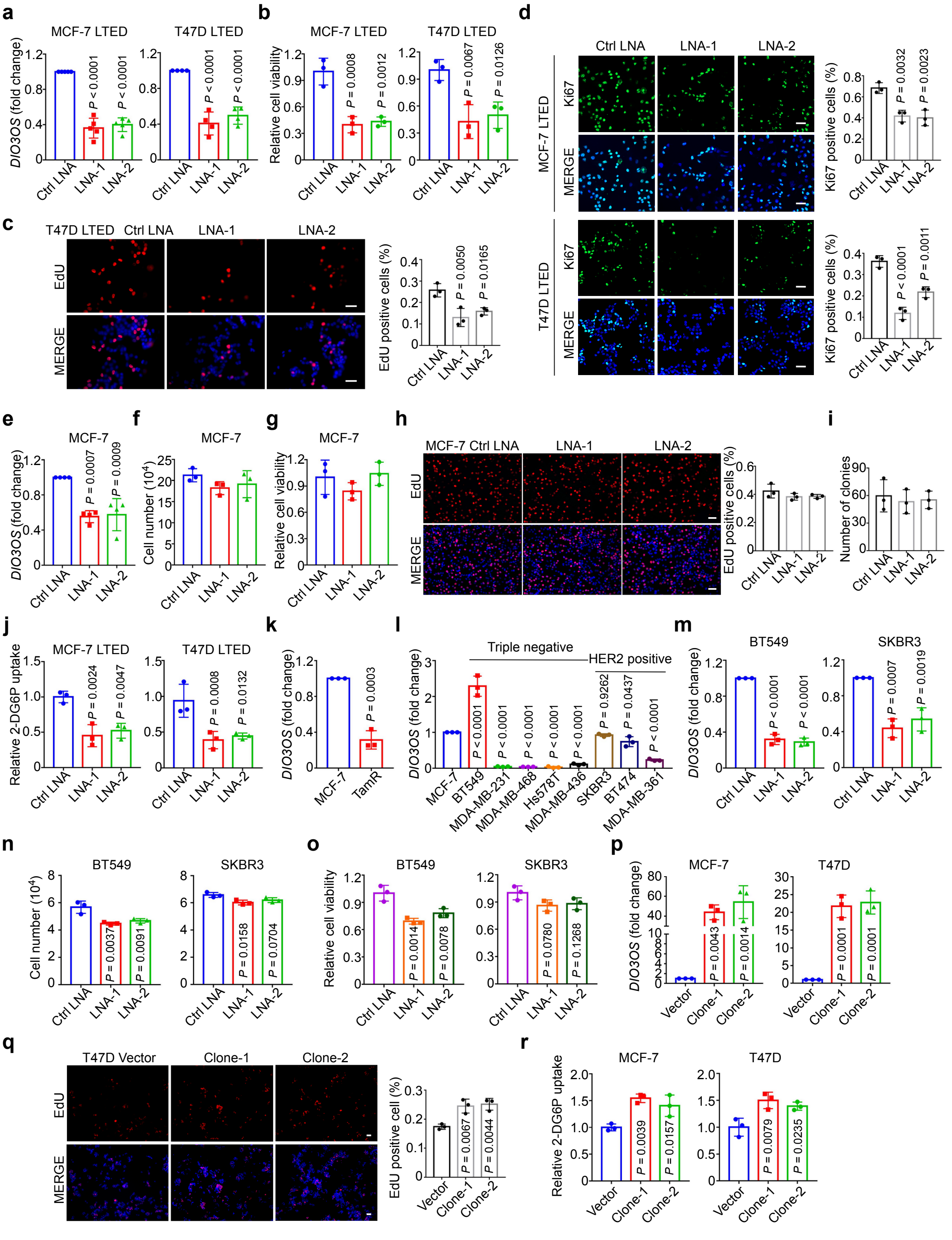

Supplementary Fig. 3 | DIO3OS regulates the proliferation and aerobic glycolysis of LTED cells.

a, qRT–PCR of DIO3OS knockdown efficiency in MCF-7/T47D LTED cells by indicated LNAs. Means ± s.d. of n = 5 (MCF-7 LTED cells) or n = 4 (T47D LTED cells) independent experiments are shown. **b**, MTT assay of MCF-7/T47D LTED transfected with indicated LNAs. **c**, Representative immunofluorescence images and quantification of EdU-incorporated T47D LTED cells with and without DIO3OS knockdown. Scale bars, 50 μm. **d**, Representative immunofluorescence images and quantification of Ki67-stained MCF-7/T47D LTED cells transfected with indicated LNAs. Scale bars, 50 μm. **e**, qRT–PCR of DIO3OS knockdown efficiency in parental MCF-7 cells by indicated LNAs. **f**,**g**, Cell number counting (**f**) and MTT assay (**g**) of parental MCF-7 cells transfected with indicated LNAs. **h**, Representative immunofluorescence images and quantification of EdU-incorporated parental MCF-7 cells with and without DIO3OS knockdown. Scale bars, 50 μm. **i**, Quantification of plate clone formation of parental MCF-7 cells transfected with indicated LNAs. **j**, Glucose uptake of MCF-7/T47D LTED cells transfected with indicated LNAs. **k**, qRT–PCR of DIO3OS expression in tamoxifen-resistant MCF-7 cells and their parental cells. **l**, qRT–PCR of DIO3OS expression in MCF-7 cells, triple negative and HER2-positive breast cancer cell lines. **m**, qRT–PCR of DIO3OS knockdown efficiency in BT549/SKBR3 cells by indicated LNAs. **n**,**o**, Cell counting (**n**) and MTT assay (**o**) of BT549/SKBR3 cells with and without DIO3OS knockdown. **p**, qRT–PCR of DIO3OS overexpression efficiency in parental MCF-7/T47D cells. **q**, Representative immunofluorescence images and quantification of EdU-incorporated parental T47D cells with and without DIO3OS overexpression. Scale bars, 50 μm. **r**, Glucose uptake of parental MCF-7/T47D cells with and without DIO3OS overexpression. Means ± s.d. of n = 3 (**b**-**d**,**f**-**r**) or n = 4 (**e**) independent experiments yielding similar results are presented, and all *P* values were calculated by two-sided one-way ANOVA with Dunnett's multiple-comparisons test. Source data are provided as a Source Data file.

Supplementary Fig. 4

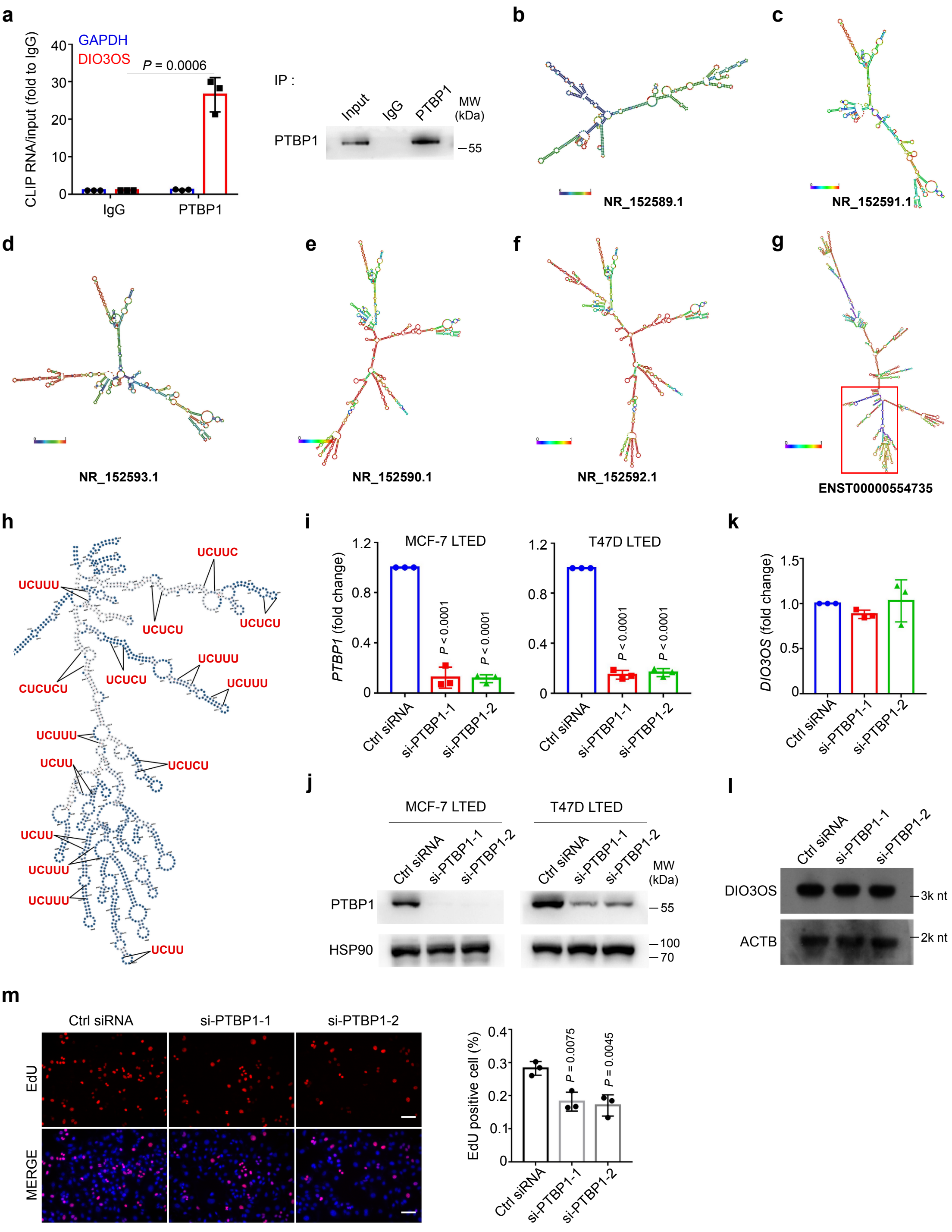

Supplementary Fig. 4 | DIO3OS interacts with PTBP1 in the nucleus.

a, CLIP assay with the anti-PTBP1 antibody demonstrating the direct interaction of DIO3OS and PTBP1 in MCF-7 LTED cells. **b-g**, Predicted secondary structures of six DIO3OS variants by RNAfold. The red box (**g**) denotes the secondary structure of ENST00000554735 differing from other five variants. **h**, Predicted PTBP1-binding motifs in the stem-loop structures surrounded by the red box in **g**. **i**,**j**, qRT–PCR (**i**) and immunoblot (**j**) of PTBP1 knockdown efficiency in MCF-7/T47D LTED cells. MW, molecular weight. **k**,**l**, qRT–PCR (**k**) and Northern blot (**l**) of DIO3OS expression in MCF-7 LTED cells with and without PTBP1 knockdown. ACTB serves as loading control RNA. nt, nucleotide. **m**, Representative immunofluorescence images and quantification of EdU-incorporated MCF-7 LTED cells transfected with control or PTBP1 siRNAs. Scale bars, 50 µm. Means ± s.d. of n = 3 (**a**,**i**,**k**,**m**) independent experiments yielding similar results, one representative experiment out of three that were similar (**j**,**l**) are shown, and *P* values were determined using two-tailed Student's *t*-test (**a**), two-sided one-way ANOVA with Dunnett's multiple-comparisons test (**i**,**k**,**m**). Source data are provided as a Source Data file.

Supplementary Fig. 5

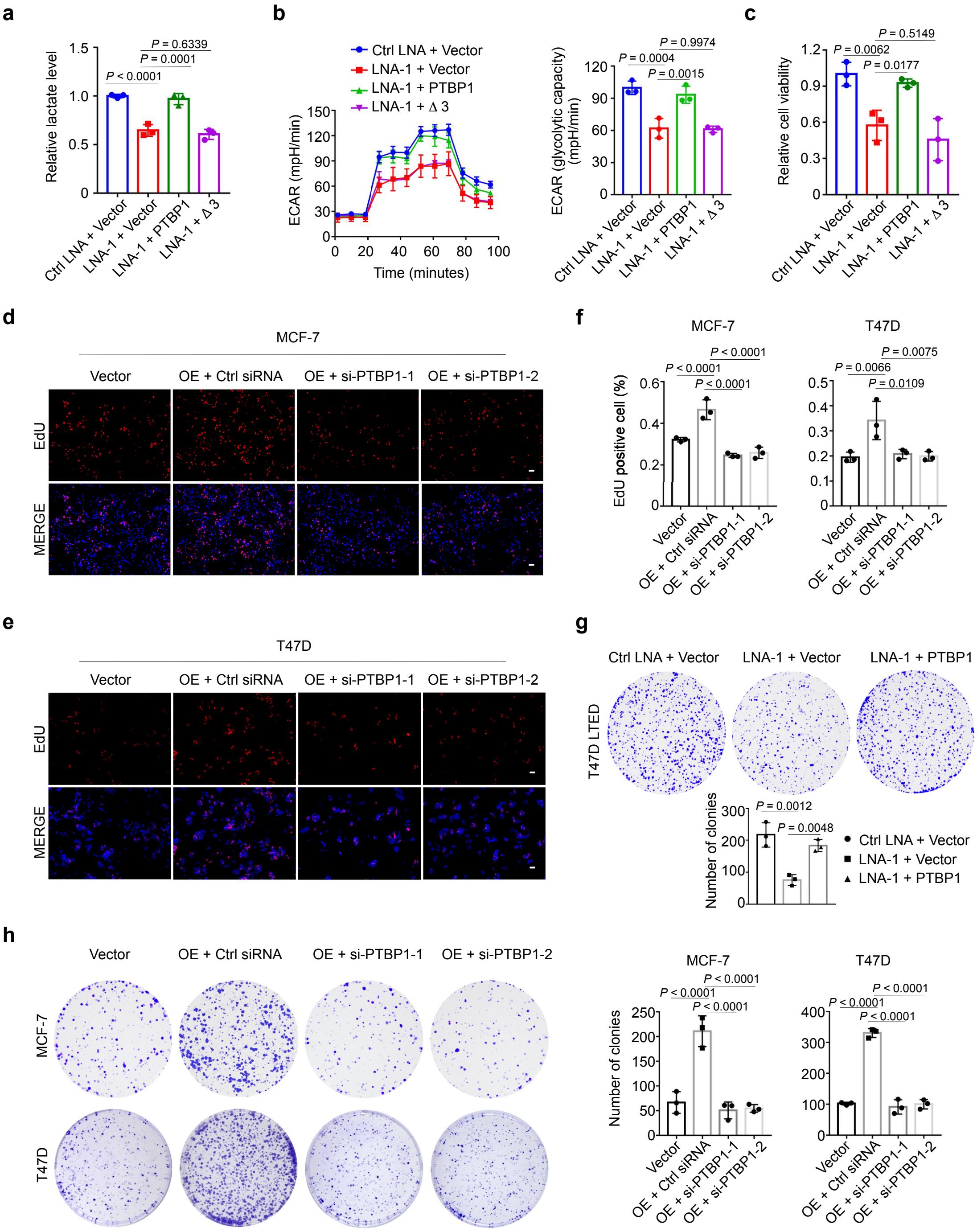

Supplementary Fig. 5 | DIO3OS regulates LTED cell proliferation and glycolysis through PTBP1.

a, Lactate production of MCF-7 LTED cells co-transfected with indicated LNAs and plasmids. **b**, ECAR values and calculated glycolytic capacity of MCF-7 LTED cells co-transfected with indicated LNAs and plasmids. **c**, MTT assay MCF-7 LTED cells co-transfected with indicated LNAs and plasmids. **d**,**e**, Representative immunofluorescence images of EdU-incorporated parental MCF-7/T47D cells transfected with control construct or co-transfected with DIO3OS expression plasmid and control or PTBP1 siRNAs. Scale bars, 50 µm. **f**, Quantification of EdU-incorporated parental MCF-7/T47D cells in **d** and **e**. **g**, Representative images and quantification of plate clone formation of T47D LTED cells co-transfected with indicated LNAs and plasmids. **h**, Representative images and quantification of plate clone formation of parental MCF-7/T47D cells with indicated plasmids and siRNAs. Means ± s.d. of n = 3 (**a**-**c**,**f**-**h**) independent experiments yielding similar results are presented, and all *P* values were analyzed using two-sided one-way ANOVA with Dunnett's multiple-comparisons test. Source data are provided as a Source Data file.

Supplementary Fig. 6

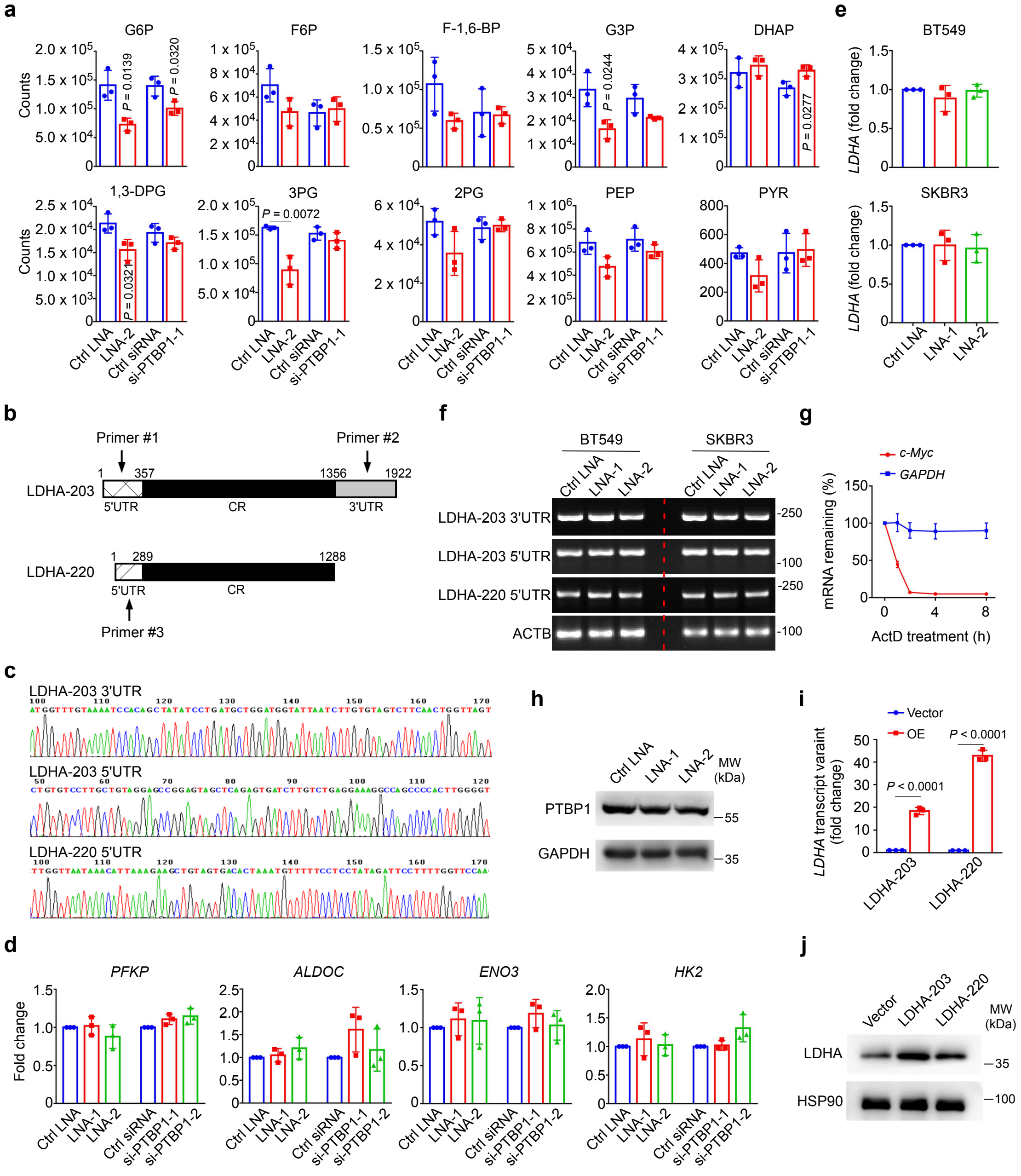

Supplementary Fig. 6 | DIO3OS and PTBP1 stabilizes LDHA mRNA by regulating its splicing switch.

a, LC-MS analysis of glycolytic intermediate production in MCF-7 LTED cells with and without DIO3OS/PTBP1 knockdown. **b**, Schematic diagram of the primers targeting the 5'/3'UTR of two LDHA variants. Nucleotide positions of the LDHA 5'/3'UTR are indicated. **c**, Sanger sequencing of the PCR products purified by gel extraction in Figure 5**f**. **d**, qRT-PCR of four glycolysis-related candidate genes in MCF-7 LTED cells with and without DIO3OS/PTBP1 knockdown. **e**, qRT-PCR of LDHA expression in BT549/SKBR3 cells with and without DIO3OS knockdown. **f**, qRT-PCR and agarose gel of the 5'/3'UTR of LDHA mRNA in BT549/SKBR3 cells with and without DIO3OS knockdown. **g**, Half-life of c-Myc and GAPDH mRNAs in MCF-7 LTED cells determined by using 10 μg/mL actinomycin D (ActD) at indicated time points. **h**, Immunoblot of PTBP1 expression in MCF-7 LTED cells with and without DIO3OS knockdown. **i**, qRT–PCR of LDHA-203/LDHA-220 overexpression efficiency in parental MCF-7 cells. **j**, Immunoblot of LDHA overexpression efficiency in parental MCF-7 cells transfected with LDHA-203/LDHA-220 plasmids. Means ± s.d. of n = 3 (**a**,**d**,**e**,**g**,**i**) independent experiments yielding similar results, one representative experiment out of three that were similar (**f**,**h**,**j**) are shown, and *P* values were calculated using two-tailed Student's *t*-test (**a**,**i**), two-sided one-way ANOVA with Dunnett's multiple-comparisons test (**d**,**e**). MW, molecular weight. Source data are provided as a Source Data file.

Supplementary Table 1 | Differential lncRNA expression in the RNA-sequencing as in Fig. 1h.

AI-resistant vs AI-sensitive	Differentially expressed lncRNA	
	**Up-regulated**	**Down-regulated**
1	LINC00989	AC107208.1
2	SH3RF3-AS1	HRAT17
3	AL596442.2	AC005699.1
4	MGC12916	AL353803.1
5	FRG1-DT	LINC01543
6	AP001476.1	AL035412.1
7	LINC01770	AL160291.1
8	C18orf65	AL034347.1
9	LINC02256	AC124276.2
10	LINC00310	AL591468.1
11	LINC01140	AC027013.1
12	LINC00840	AC011816.2
13	DIO3OS	AC005899.7
14	AC025035.1	AC055811.3
15	AP000442.1	MMP2-AS1
16	AL118556.1	LINC00943
17	AC068790.9	AC092681.2
18	DPH6-DT	AC125494.2
19	LINC00908	ERVH48-1
20	LINC01474	AC017002.3
21	AC005180.1	AC112721.2
22	AL050327.1	AC092803.2
23	LINC01356	AC104785.1
24	LINC01264	AC126773.4
25	AC004947.1	LINC01943
26	AC024257.5	AP002360.2
27	LINC01230	AL031123.1
28	AC002546.1	
29	PGM5P3-AS1	
30	AC016924.1	
31	AC013448.1	
32	AL365181.4	
33	LINC02544	

Supplementary Table 2 | Correlation of DIO3OS expression with the clinicopathological status in 257 cases of AI-treated ER-positive breast cancer patients.

	No. of patients	DIO3OS[#]		*P* value
		< 3	≥ 3	
**Tumor Size (cm)**				
≤2	121	78 (64.5%)	43 (35.5%)	0.014*
> 2	136	67 (49.3%)	69 (50.7%)	
**Histological Grade**				
I	24	16 (66.7%)	8 (33.3%)	0.329
II	132	77 (58.3%)	55 (41.7%)	
III	101	52 (51.5%)	49 (48.5%)	
**Lymph Node Metastasis**				
0	109	61 (56.0%)	48 (44.0%)	0.238
1~3	66	43 (65.2%)	23 (34.8%)	
4~9	38	21 (55.3%)	17 (44.7%)	
≥ 10	44	20 (45.5%)	24 (54.5%)	
**Distant organ metastasis**				
Negative	206	120 (58.2%)	86 (41.7%)	0.234
Positive	51	25 (49.0%)	26 (51.0%)	
**Stage**				
Tis	7	4 (57.1%)	3 (42.9%)	0.448
I	60	36 (60.0%)	24 (40.0%)	
II	83	52 (62.7%)	31 (37.3%)	
III	56	28 (50.0%)	28 (50.0%)	
IV	51	25 (49.0%)	26 (51.0%)	
**Ki-67 (% of tumor cells)**				
≤14%	65	48 (73.8%)	17 (26.2%)	0.001*
> 14%	192	97 (50.5%)	95 (49.5%)	

[#] DIO3OS expression in breast cancer tissue was determined by ISH and cut off by X-tile analysis.

* $P < 0.05$, statistically significant and determined by two-tailed $\chi 2$ test.

Supplementary Table 3 | Proteins pulled by DIO3OS in MCF-7 LTED cells.

	Protein description	Score	Matches	Sequences	emPAI
1	Polypyrimidine tract-binding protein 1	1387	80(52)	29(21)	5.66
2	Keratin, type II cytoskeletal 1	855	57(37)	28(24)	2.53
3	Keratin, type I cytoskeletal 10	784	54(28)	26(20)	2.48
4	Keratin, type II cytoskeletal 2 epidermal	770	47(28)	26(18)	1.93
5	60S ribosomal protein L4	760	71(41)	31(21)	5.88
6	Polypyrimidine tract-binding protein 3	561	43(26)	18(13)	1.62
7	60S ribosomal protein L6	392	35(17)	20(12)	3.26
8	Double-stranded RNA-binding protein Staufen homolog 1	350	37(20)	21(16)	1.48
9	60S ribosomal protein L7	303	36(14)	19(11)	3.05
10	60S ribosomal protein L13	261	25(12)	10(8)	2.19
11	60S ribosomal protein L7a	234	21(11)	15(10)	1.84
12	Keratin, type II cytoskeletal 5	204	21(9)	14(7)	0.59
13	Serum albumin	173	15(7)	7(5)	0.31
14	Keratin, type I cytoskeletal 14	170	17(8)	10(6)	0.64
15	Keratin, type I cytoskeletal 9	169	18(8)	12(7)	0.43
16	RNA-binding protein 34	164	10(4)	8(4)	0.30
17	Keratin, type I cytoskeletal 18	154	3(3)	2(2)	0.14
18	60S ribosomal protein L3	149	25(9)	14(7)	0.62
19	Tubulin alpha-1C chain	130	9(4)	6(3)	0.29
20	60S ribosomal protein L8	119	14(8)	11(6)	0.95
21	60S ribosomal protein L29	113	8(4)	5(3)	1.01
22	Keratin, type II cytoskeletal 8	112	12(5)	7(3)	0.35
23	40S ribosomal protein S2	76	3(3)	3(3)	0.35
24	60S acidic ribosomal protein P0	75	11(5)	7(4)	0.58
25	40S ribosomal protein S4, X isoform	67	10(3)	8(3)	0.37
26	Transthyretin	65	2(2)	1(1)	0.47
27	Putative elongation factor 1-alpha-like 3	64	5(1)	2(1)	0.07
28	60S ribosomal protein L22	62	1(1)	1(1)	0.23
29	Serine beta-lactamase-like protein LACTB, mitochondrial	62	12(4)	8(3)	0.30
30	60S ribosomal protein L18	60	3(2)	3(2)	0.33
31	Tubulin beta-2B chain	56	3(1)	3(1)	0.14
32	60S ribosomal protein L10a	54	4(1)	4(1)	0.13
33	40S ribosomal protein S6	54	11(3)	9(2)	0.24
34	60S ribosomal protein L21	45	5(2)	4(2)	0.39
35	40S ribosomal protein S8	43	6(1)	5(1)	0.14
36	Serpin A12	41	10(2)	1(1)	0.07
37	Y-box-binding protein 3	40	14(3)	12(3)	0.27
38	Hornerin	36	4(2)	2(1)	0.02
39	2'-5'-oligoadenylate synthase-like protein	34	1(1)	1(1)	0.06
40	60S ribosomal protein L10	34	5(1)	3(1)	0.13
41	Heterogeneous nuclear ribonucleoprotein H2	33	6(2)	5(2)	0.14
42	Transmembrane and coiled-coil domain-containing protein 3	31	3(3)	1(1)	0.04

Supplementary Table 4 | MS/MS of human PTBP1 peptide (57221Dal). Related to Fig. 3 and Supplementary Table 3.

Query	Observed	Mr(expt)	Mr(calc)	ppm	Score	Expect	Rank	Peptide
26	361.7260	721.4375	721.4374	0.14	29	0.0017	1	K.IITFTK.N
149	381.7486	761.4826	761.4799	3.56	42	0.00019	1	K.ILFNKK.E
173	383.2599	764.5052	764.5021	4.12	16	0.028	2	R.VIHIRK.L
221	387.7406	773.4666	773.4647	2.45	37	0.0034	1	K.LTSLNVK.Y
453	410.7628	819.5110	819.5079	3.83	22	0.014	1	K.LHGKPIR.I
554	419.2333	836.4521	836.4504	2.02	44	0.00032	1	K.KFKGDSR.S
1001	451.2523	900.4900	900.4858	4.65	10	2.9	1	K.GFKFFQK.D
1209	466.2871	930.5595	930.5572	2.52	48	0.00012	1	K.VTNLLMLK.G
1326	474.2855	946.5564	946.5521	4.48	(42)	0.00048	1	K.VTNLL->M<-LK.G
1460	484.7698	967.5250	967.5239	1.10	12	0.65	4	K.FFQKDRK.M
1649	496.2764	990.5382	990.5359	2.31	54	5.4e-005	1	K.HQNVQLPR.E
2125	529.7545	1057.4944	1057.4941	0.26	(59)	1.1e-005	1	K.DYGNSPLHR.F
2126	353.5064	1057.4972	1057.4941	2.96	59	1e-005	1	K.DYGNSPLHR.F
2501	553.8175	1105.6204	1105.6132	6.58	68	2e-006	1	K.VLFSSNGGVVK.G
4276	682.8962	1363.7779	1363.7711	5.00	(54)	2.6e-005	1	R.IDFSKLTSLNVK.Y
4280	455.6003	1363.7792	1363.7711	5.93	57	1.4e-005	1	R.IDFSKLTSLNVK.Y
4560	470.2552	1407.7439	1407.7358	5.80	1	11	1	K.LTSLNVKYNNDK.S
4732	716.3769	1430.7392	1430.7306	6.00	54	8.6e-005	1	R.GQPIYIQFSNHK.E
4734	477.9204	1430.7393	1430.7306	6.06	(44)	0.00086	1	R.GQPIYIQFSNHK.E
5311	511.9688	1532.8844	1532.8787	3.74	6	1.2	1	R.ITLSKHQNVQLPR.E
6461	601.3280	1800.9622	1800.9522	5.51	40	0.0016	1	R.GQPIYIQFSNHKELK.T
6465	451.2481	1800.9633	1800.9522	6.16	(27)	0.036	1	R.GQPIYIQFSNHKELK.T
6855	949.4514	1896.8883	1896.8822	3.21	93	5.8e-009	1	K.LSLDGQNIYNACCTLR.I
6858	633.3042	1896.8908	1896.8822	4.52	(45)	0.00037	1	K.LSLDGQNIYNACCTLR.I
7259	992.5577	1983.1008	1983.0929	4.01	40	0.00075	1	K.LPIDVTEGEVISLGLPFGK.V
7323	500.5193	1998.0481	1998.0422	2.94	37	0.0029	1	R.IDFSKLTSLNVKYNNDK.S
7461	680.3743	2038.1012	2038.0888	6.07	65	3.2e-006	1	R.VTPQSLFILFGVYGDVQR.V
7462	1020.0593	2038.1041	2038.0888	7.52	(44)	0.00045	1	R.VTPQSLFILFGVYGDVQR.V
7581	693.0231	2076.0474	2076.0348	6.05	15	0.47	1	K.HQNVQLPREGQEDQGLTK.D
7744	704.7412	2111.2018	2111.1878	6.63	71	3.4e-007	1	R.KLPIDVTEGEVISLGLPFGK.V
7747	1056.6130	2111.2115	2111.1878	11.2	(33)	0.0017	1	R.KLPIDVTEGEVISLGLPFGK.V
7899	1072.5084	2143.0023	2142.9930	4.33	(35)	0.0035	1	R.EGQEDQGLTKDYGNSPLHR.F
7900	715.3417	2143.0034	2142.9930	4.84	80	1.2e-007	1	R.EGQEDQGLTKDYGNSPLHR.F
7905	536.7588	2143.0061	2142.9930	6.09	(54)	5.2e-005	1	R.EGQEDQGLTKDYGNSPLHR.F
8411	561.5347	2242.1098	2242.1131	-1.44	(6)	3.9	1	K.NNQFQALLQYADPVSAQHAK.L
8412	1122.0630	2242.1114	2242.1131	-0.72	(26)	0.043	1	K.NNQFQALLQYADPVSAQHAK.L
8414	748.3812	2242.1219	2242.1131	3.93	68	2.5e-006	1	K.NNQFQALLQYADPVSAQHAK.L
8572	759.1026	2274.2860	2274.2695	7.22	(56)	8.8e-006	1	R.IAIPGLAGAGNSVLLVSNLNPER.V
8573	1138.1511	2274.2877	2274.2695	7.98	70	3.6e-007	1	R.IAIPGLAGAGNSVLLVSNLNPER.V
10311	690.3565	2757.3967	2757.3834	4.82	9	1.8	1	R.GQPIYIQFSNHKELKTDSSPNQAR.A
10957	737.3972	2945.5598	2945.5399	6.74	21	0.078	1	K.IITFTKNNQFQALLQYADPVSAQHAK.L
11056	998.8534	2993.5385	2993.5134	8.38	14	0.39	1	K.NFQNIFPPSATLHLSNIPPSVSEEDLK.V

Supplementary Table 5 | Primers used in the qRT-PCR and PCR assays for indicated genes, related to methods.

**Gene name**	**Sequence**
**qRT-PCR**	
GAPDH	5’-ATCACCATCTTCCAGGAGCGA-3’(forward)
	5’-CCTTCTCCATGGTGGTGAAGAC-3’(reverse)
ACTB	5’-GATGCGTTGTTACAGGAAGTCC-3’(forward)
	5’-GGCACGAAGGCTCATCATTCA-3’(reverse)
DIO3OS	5’-AGGCCCAGCCCAATAGGAA-3’(forward)
	5’-GGCCCAAGAAACAGCAACA-3’(reverse)
ESR1	5’-GAAAGGTGGGATACGAAAAGACC-3’(forward)
	5’-GCTGTTCTTCTTAGAGCGTTTGA-3’(reverse)
AC004947	5’-GGCTCGGCAGAGAACTCAGTGA-3’(forward)
	5’-AGGCGGGCAATGAAGGGCTT-3’(reverse)
AC002546	5’-GCAGCCTTCCAATCCAGCAGAG-3’(forward)
	5’-GGAGAAGTCTGAGGGCAGGAGTC-3’(reverse)
LINC01230	5’-GGACTGGCTGTGCTGCTACCT-3’(forward)
	5’-TCAGTTGCCTCAGTTGCCTCATC-3’(reverse)
LDHA	5’-TTGACCTACGTGGCTTGGAAG-3’(forward)
	5’-GGTAACGGAATCGGGCTGAAT-3’(reverse)
LDHA-203 3'UTR	5’-AGGTGGAGGTTGTGCATGTTG-3’(forward)
	5’-GCAGTACGTACAGCATTGGCA-3’(reverse)
LDHA-203 5'UTR	5’-TCGGAGGACCCAGCAATTAGTC-3’(forward)
	5’-TACTCCGGCTCCTACAGCAAG-3’(reverse)
LDHA-220 5'UTR	5’-AGTGGCAAATACCCCCAAAGGA-3’(forward)
	5’-ATTCTGGGGGGTCTGTTCTTCC-3’(reverse)
Luciferase	5’-GATTGACAAGGATGGATGGC-3’(forward)
	5’-CGTCATCGTCGGGAAGACCT-3’(reverse)
MALAT1	5’-GTCATAACCAGCCTGGCAGT-3’(forward)
	5’-CGAAACATTGGCACACAGCA-3’(reverse)
PTBP1	5’-AGCGCGTGAAGATCCTGTTC-3’(forward)
	5’-CAGGGGTGAGTTGCCGTAG-3’(reverse)
PFKP	5’-CGCCTACCTCAACGTGGTG-3’(forward)
	5’-ACCTCCAGAACGAAGGTCCTC-3’(reverse)
ALDOC	5’-ATGCCTCACTCGTACCCAG-3’(forward)
	5’-TTTCCACCCCAATTTGGCTCA-3’(reverse)
ENO3	5’-TATCGCAATGGGAAGTACGATCT-3’(forward)
	5’-AAGCTCTTATACAGCTCTCCGA-3’(reverse)
HK2	5’-TTGACCAGGAGATTGACATGGG-3’(forward)
	5’-CAACCGCATCAGGACCTCA-3’(reverse)
c-Myc	5’-GTCAAGAGGCGAACACACAAC-3’(forward)

	5’-TTGGACGGACAGGATGTATGC-3’(reverse)
**PCR**	
LDHA-220 5’UTR	5’-CCTGTCATTAGGCCTTTCAACT-3’(forward)
	5’-TGCCATATTGGACTTGGAACC-3’(reverse)
LDHA-203 3'UTR	5’-GGCTACAACAGGATTCTAGGTG-3’(forward)
	5’-CACACGGTAAACATCCACCT-3’(reverse)
LDHA-203 5'UTR	5’-TGCCTTGGGCTTGAGCTTTG-3’(forward)
	5’-GGTTCACCCATCGCGGTTTAT-3’(reverse)
ACTB	5’-GATGCGTTGTTACAGGAAGTCC-3’(forward)
	5’-GGCACGAAGGCTCATCATTCA-3’(reverse)
**RACE**	
3’GSP_F1	5’-CGTCAAGCCCAGCCCAATAGGAAGCACCTG-3’ (forward)
3’GSP_F2	5’-ACAGAATACACTCCACACCTCGGGACTCCA-3’ (forward)
3’GSP_F3	5’-TGGAGGAGAGTGCTGGTGAATGGCAGGTGT-3’ (forward)
3’GSP_F4	5’-AGGTGTGTGCCTCAGTGTCCTTGCTGCCA-3’ (forward)
5’GSP_R1	5’-GGTCCCTGCCTGGTGCTGGTTTGGGTTAGT-3’(reverse)
5’GSP_R2	5’-ACACCTGCCATTCACCAGCACTCTCCTCCA-3’ (reverse)
5’GSP_R3	5’-TGTAAGAGGGCAGGCAGAAAGGGAGGTGGG-3’ (reverse)
5’GSP_R4	5’-CCCTCCTGACCACTGGGATGGGACAAGACA-3’ (reverse)
5’GSP_R5	5’-GCCTGGACCTTGGGTCTGTCCTCACCTGCA-3’(reverse)
5’GSP_R6	5’-GGCTAACTCAGAGGTGCCCTGATGGAGGGG-3’(reverse)
5’GSP_R7	5’-GCGACTGCGACAGTGTCTCGGGCAGTTCAG-3’(reverse)

Supplementary Table 6 | LNAs, siRNAs and shRNAs for cell transfection, related to methods.

**Name**	**Sequence**
**LNA**	
Ctrl-LNA	AACACGTCTATACGC
LNA-1	AGAAAGAGTGTGGATC
LNA-2	GACAAACGGCCAGTGG
**siRNA**	
Ctrl siRNA	GGGUUGUAGAGUGCAUAAATT
si-PTBP1-1	GCACAGUGUUGAAGAUCAUTT
si-PTBP1-2	CCCAAAGCCUCUUUAUUCUTT
si-ESR1-1	GAGGGAGAAUGUUGAAACATT
si-ESR1-2	GGCUAGAGAUCCUGAUGAUTT
**shRNA**	
sh-Ctrl	TTCTCCGAACGTGTCACGT
sh-DIO3OS	CAGTGACTAACCCAAACCA

Uncropped Blots and Gels

Fig. 2a

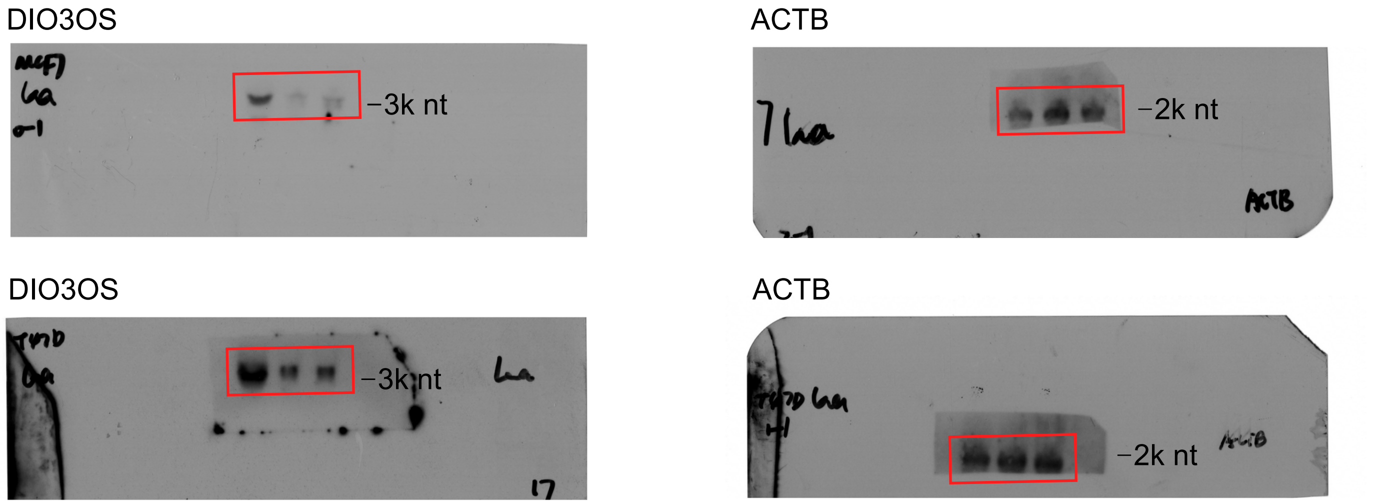

Fig. 2h

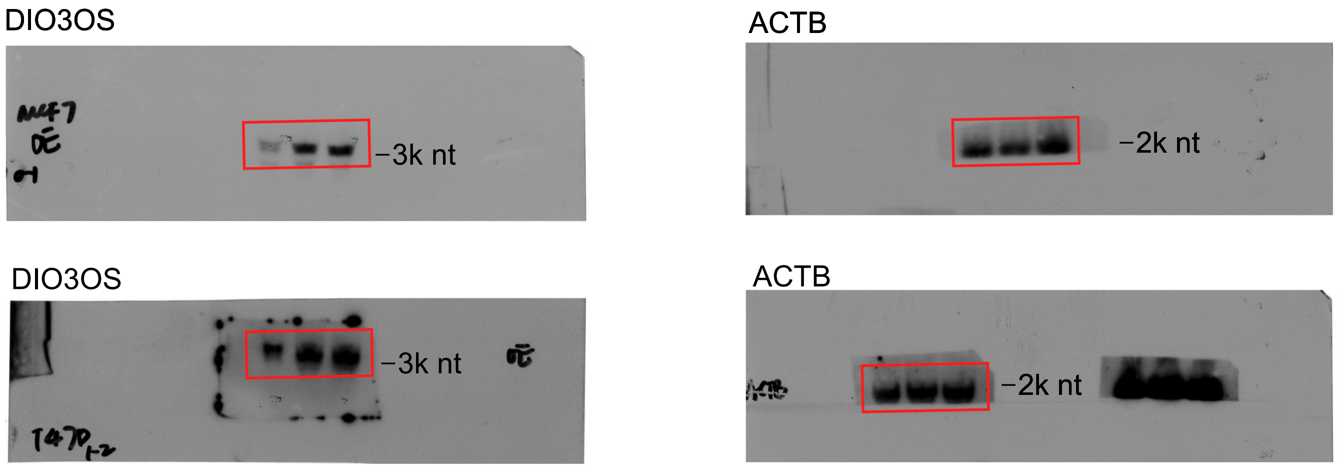

Fig. 3a

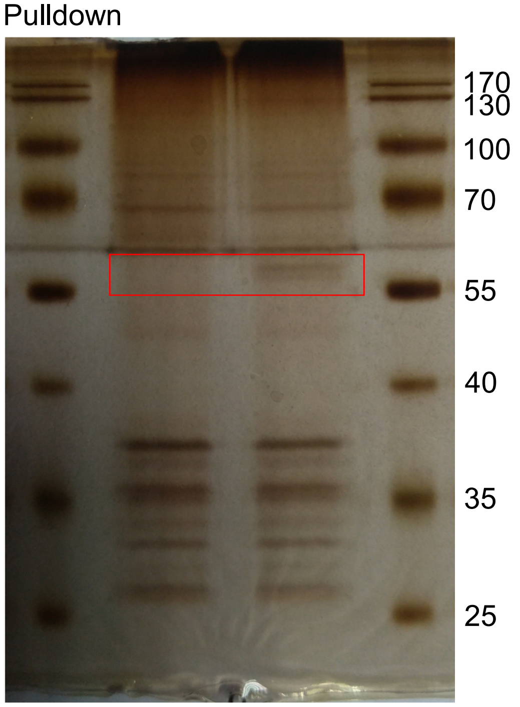

Fig. 3c

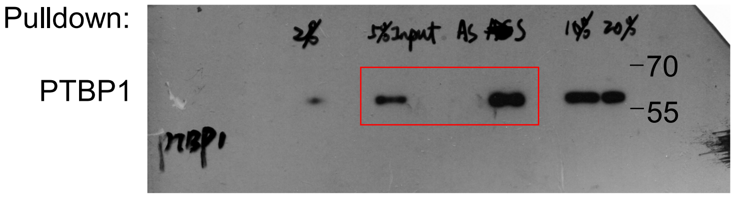

Fig. 3d

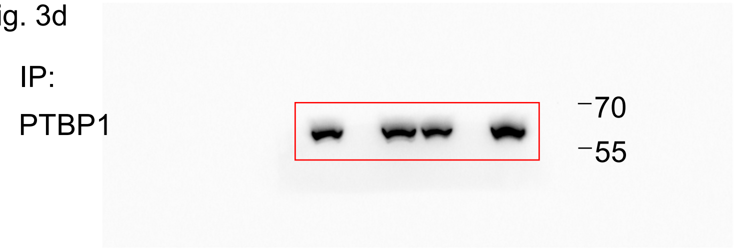

Fig. 3f

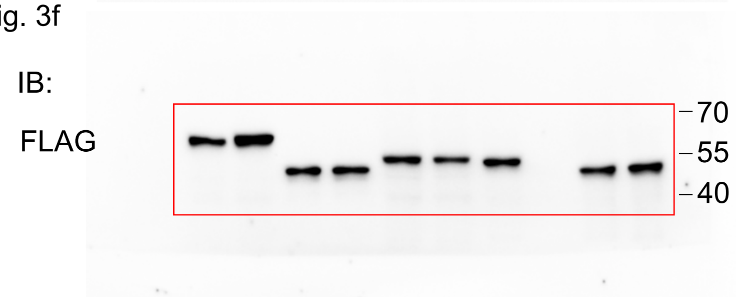

Fig. 5f

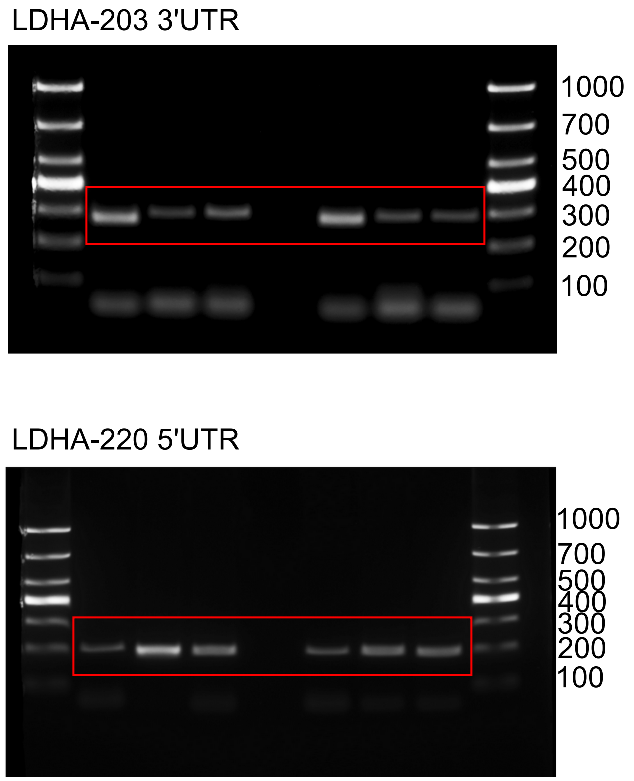

Fig. 5h

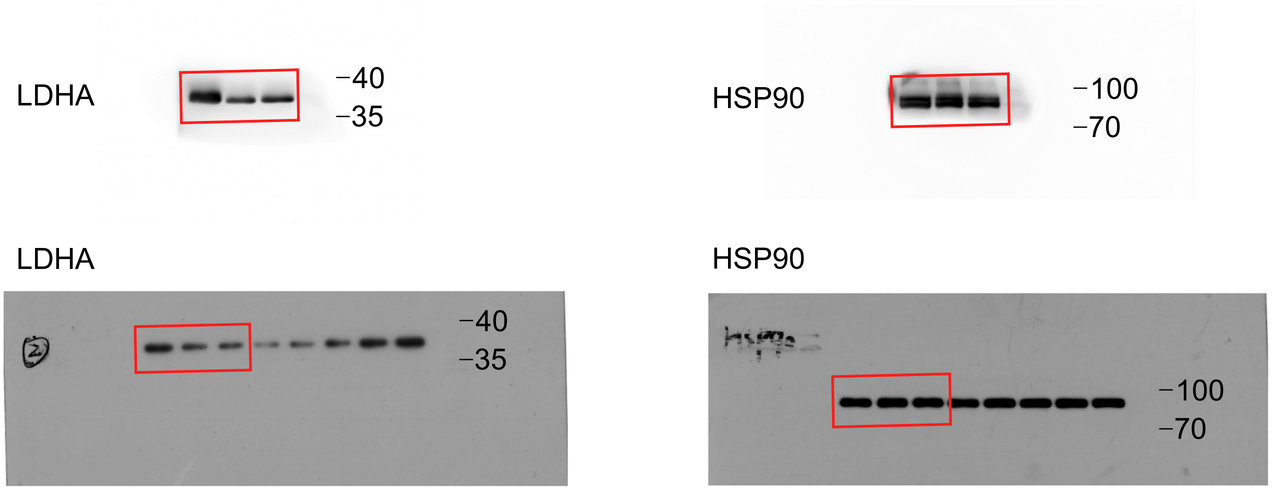

Fig. 5l

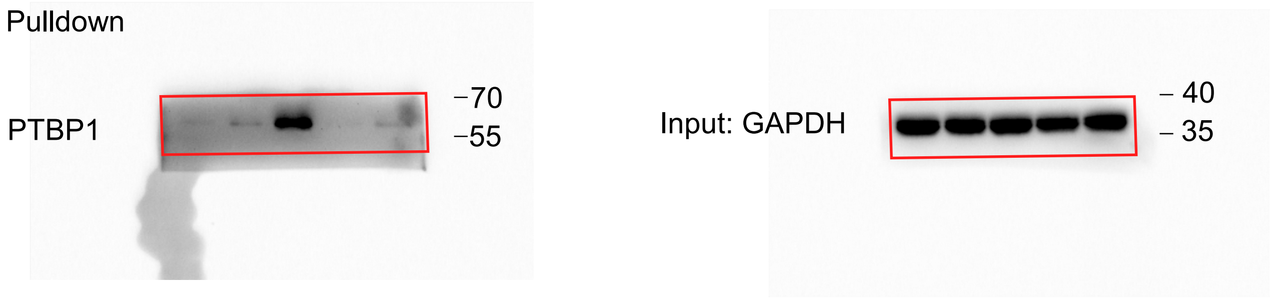

Fig. 6a

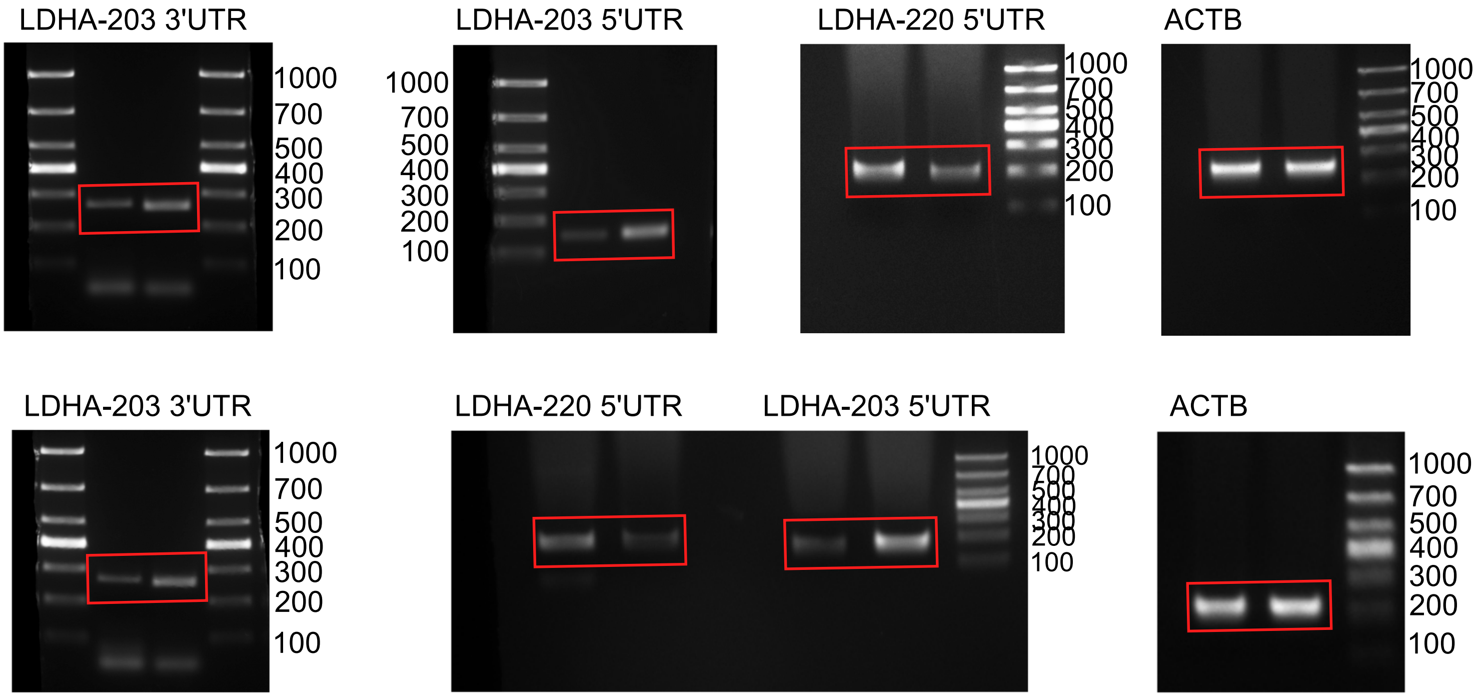

Fig. 6c

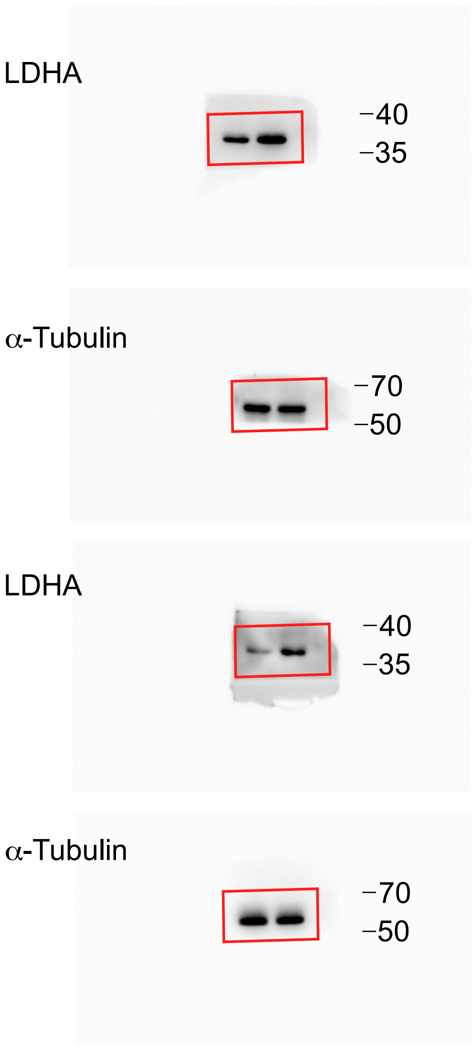

Fig. 6e

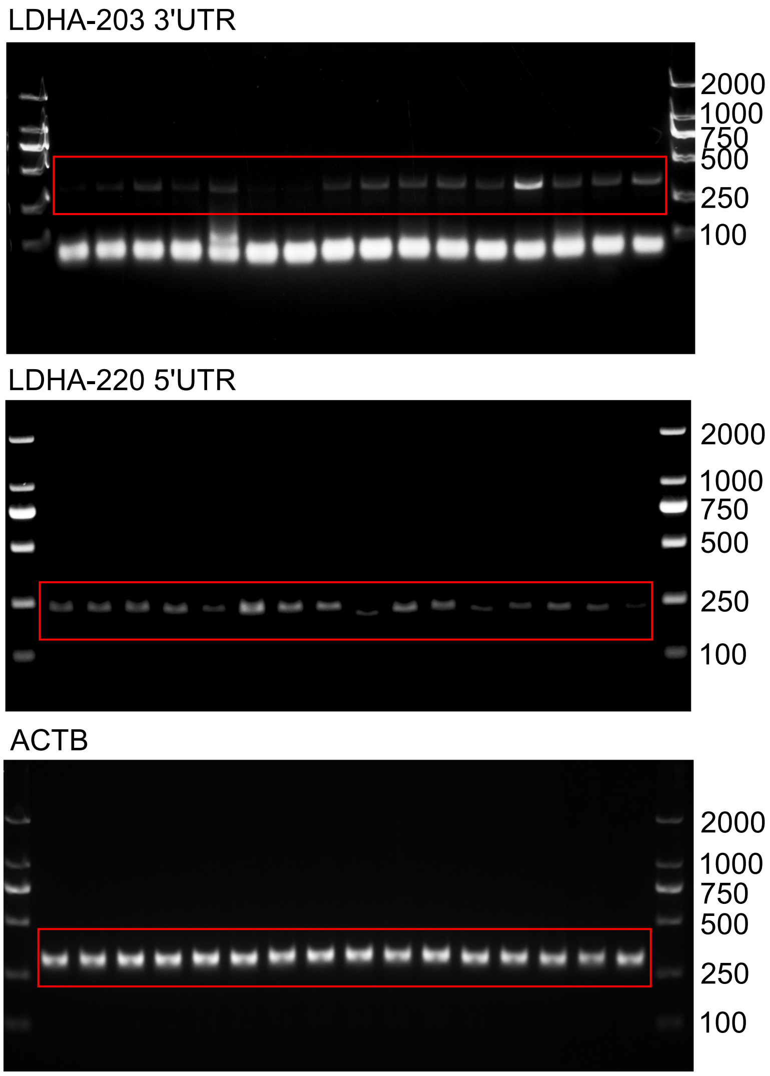

Supplementary Fig. 1j

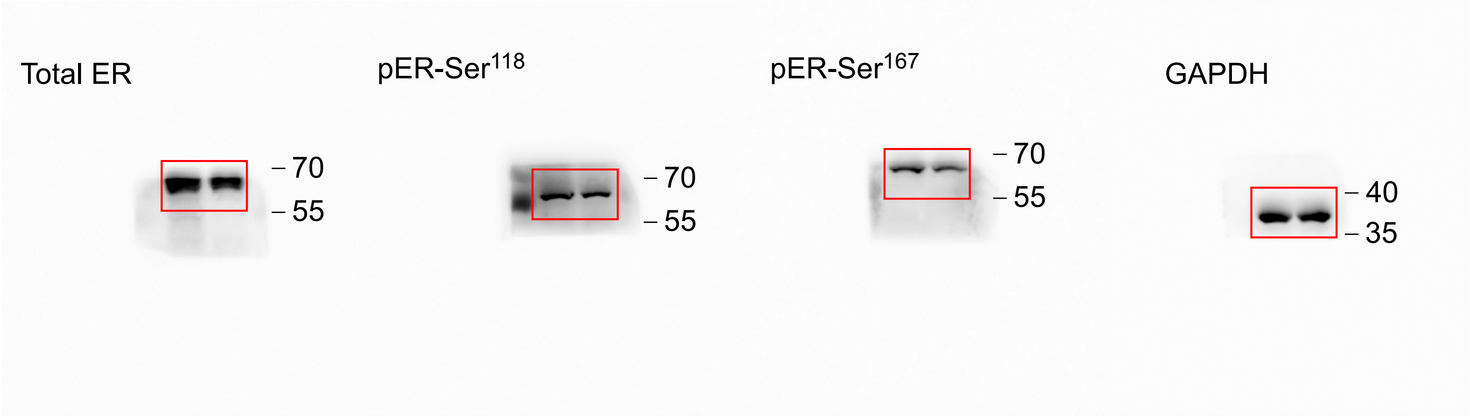

Supplementary Fig. 1m

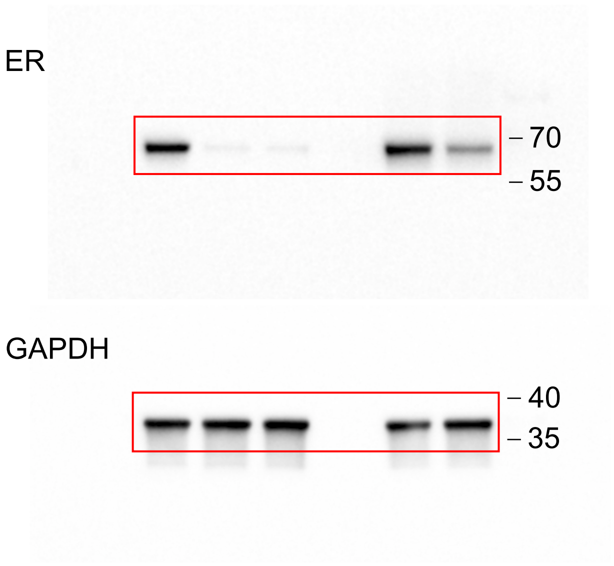

Supplementary Fig. 2b

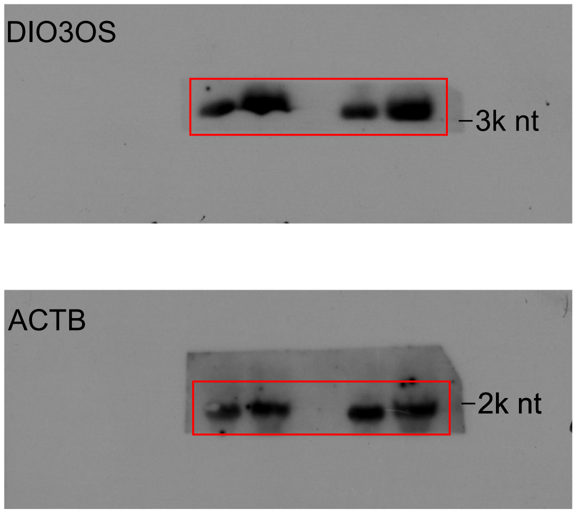

Supplementary Fig. 2c

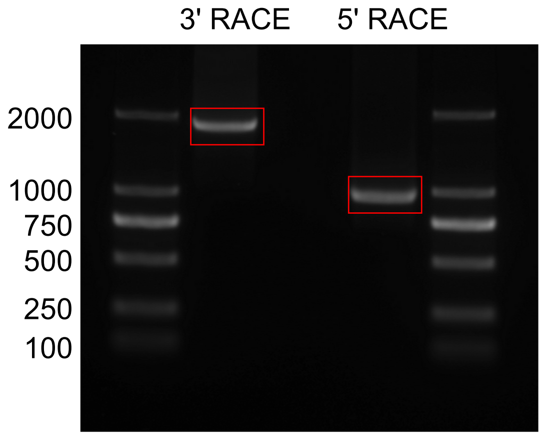

Supplementary Fig. 2e

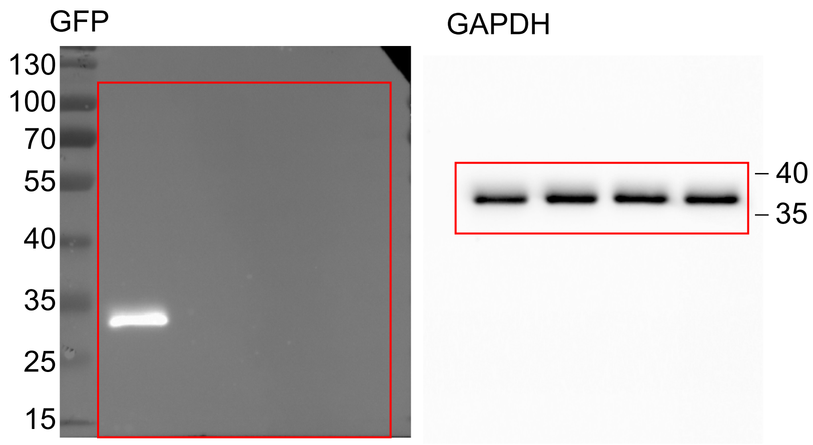

Supplementary Fig. 4a

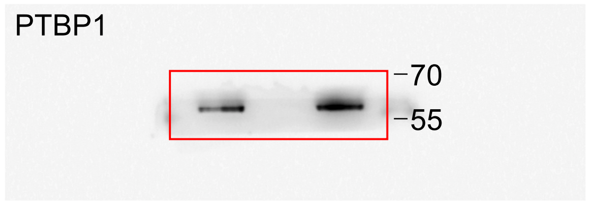

Supplementary Fig. 4j

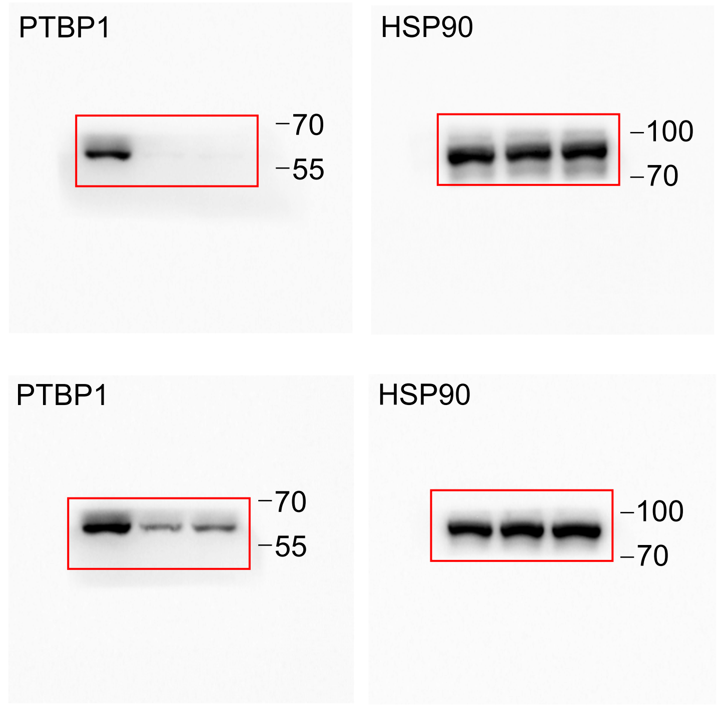

Supplementary Fig. 4l

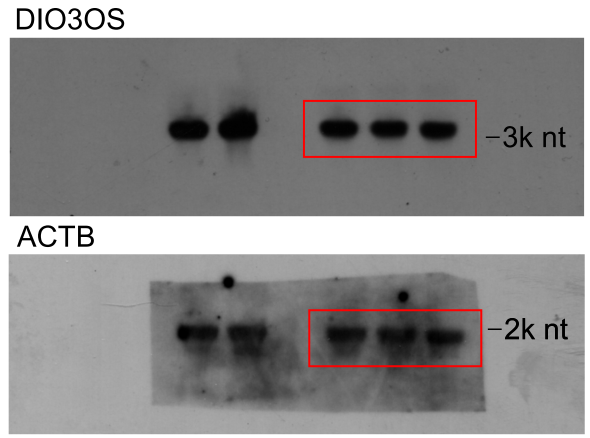

Supplementary Fig. 6f

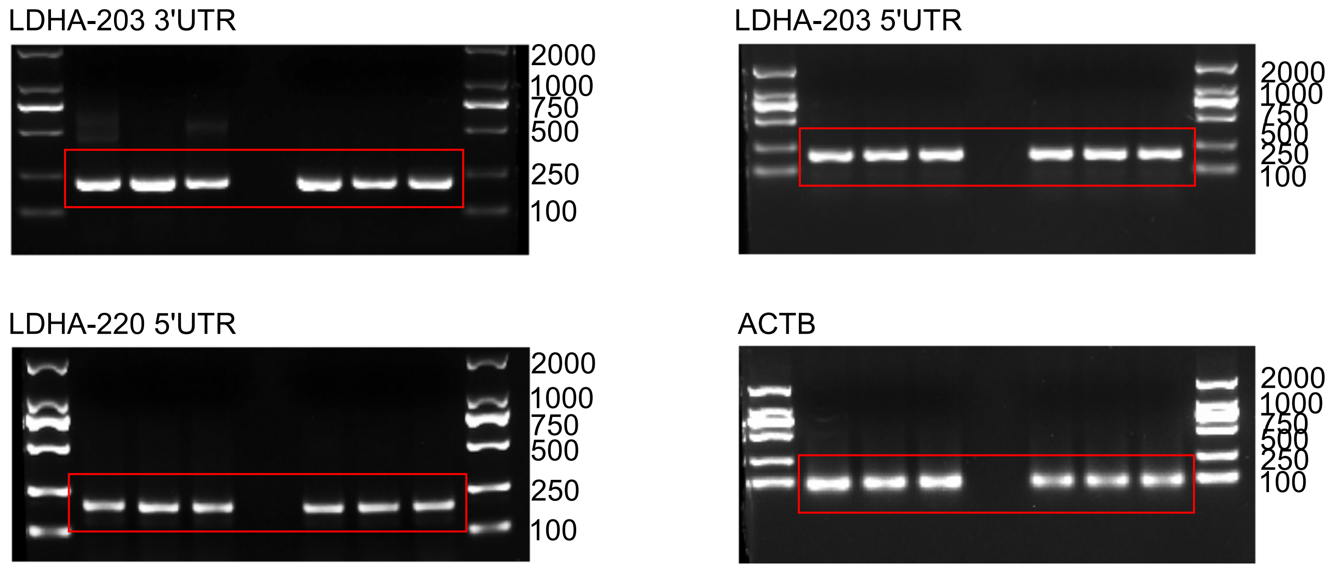

Supplementary Fig. 6h

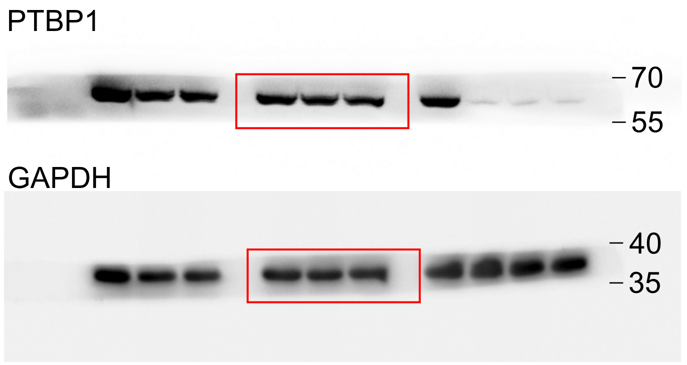

Supplementary Fig. 6j

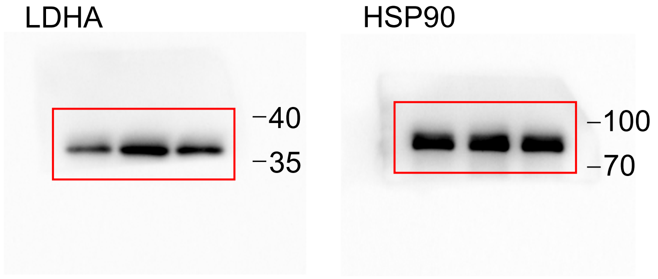

Supplementary Note 1 | Peptides (red) match to homo sapiens PTBP1 translation (531 aa):

MDGIVPDIAVGTKRGSDELFSTCVTNGPFIMSSNSASAANGNDSKKFKGDSR
SAGVPSRVIHIRKLPIDVTEGEVISLGLPFGKVTNLLMLKGKNQAFIEMNTEE
AANTMVNYYTSVTPVLRGQPIYIQFSNHKELKTDSSPNQARAQAALQAVNS
VQSGNLALAASAAAVDAGMAMAGQSPVLRIIVENLFYPVTLDVLHQIFSKF
GTVLKIITFTKNNQFQALLQYADPVSAQHAKLSLDGQNIYNACCTLRIDFSK
LTSLNVKYNNDKSRDYTRPDLPSGDSQPSLDQTMAAAFGLSVPNVHGALAP
LAIPSAAAAAAAAGRIAIPGLAGAGNSVLLVSNLNPERVTPQSLFILFGVYGD
VQRVKILFNKKENALVQMADGNQAQLAMSHLNGHKLHGKPIRITLSKHQN
VQLPREGQEDQGLTKDYGNSPLHRFKKPGSKNFQNIFPPSATLHLSNIPPSVS
EEDLKVLFSSNGGVVKGFKFFQKDRKMALIQMGSVEEAVQALIDLHNHDLG
ENHHLRVSFSKSTI
