# Supplementary material for: Long noncoding RNA DIO3OS induces glycolytic-dominant metabolic reprogramming to promote aromatase inhibitor resistance in breast cancer
Source: Nat Commun. 2022 Nov 22;13:7160. doi: 10.1038/s41467-022-34702-x (PMC9684133; doi:10.1038/s41467-022-34702-x)
Supplement: Supplementary file 2 — Description of Additional Supplementary Files [file 41467_2022_34702_MOESM2_ESM.pdf]

Description of Additional Supplementary Files

File Name: Supplementary Data 1

Description: Mass spectrometry data for all proteins pulled by DIO3OS

File Name: Supplementary Data 2

Description: Analysis of alternative splicing events identified from RNA-seq data
